# Evaluation and management of autonomic functions in patients with spinal cord injury: A scoping review

**DOI:** 10.1080/10790268.2025.2485509

**Published:** 2025-04-07

**Authors:** Ram Lochan Yadav, Jessica Catherine Martin, Mary Pauline Galea

**Affiliations:** 1 Department of Medicine (Royal Melbourne Hospital), The University of Melbourne, Parkville, Australia; 2 Department of Biomedical Engineering, The University of Melbourne, Parkville, Australia; 3 Victorian Spinal Cord Service, Austin Health, Heidelberg, Australia

**Keywords:** Spinal cord injury, Cardiovascular autonomic functions, Autonomic assessment, Autonomic management

## Abstract

**Context:**

Individuals with high-level spinal cord injury (SCI) face serious cardiovascular (CV) autonomic complications, contributing to increased mortality and morbidity. The assessment of CV autonomic function in SCI is challenging and varies widely across studies, with no clear or definitive interventions to restore hemodynamic stability or prevent complications.

**Objectives:**

This review outlines available clinical data on measuring and managing CV autonomic dysfunction after SCI and identifies gaps in these domains.

**Methods:**

A scoping review was conducted, using a search strategy targeting broad keywords related to SCI, autonomic function parameters, and management from six relevant databases, as well as reference lists and gray literatures.

**Results:**

Of 2,749 articles identified, 92 were included. Studies categorized CV autonomic complications by acute and chronic stages. High-level SCI was commonly associated with bradycardia, low BP, orthostatic hypotension, and autonomic dysreflexia, compared to lower thoracic SCI. However, the correlation between these complications and SCI completeness was unclear. Various measurement methods were used, including 24-hour ambulatory BP monitoring (ABPM), ECG derivatives, heart rate variability, sympathetic skin response, cold pressor test, head-up tilt, the International Standards to document Autonomic Function following SCI (ISAFSCI) and the Autonomic Dysfunction Following SCI (ADFSCI) tools. Of these, 24-hour ABPM demonstrated superiority in identifying diurnal variation and activity effects on CV conditions. Studies reported mixed outcomes for both pharmacological and non-pharmacological management of CV complications.

**Conclusion:**

Research gaps persist, especially in sub-acute stages and in standardized tools for assessing CV autonomic dysfunction. Chronic complications have a long-term impact on health and CV disease risk. While promising methodologies exist, such as 24-hour ABPM and questionnaire-based assessments, further refinement is needed. Comprehensive management strategies should also be developed. This includes emerging techniques like spinal neuromodulation, which require extensive research and clinical trials.

## Introduction

Cervical spinal cord injury (SCI) is one of the most life-changing and devastating events. It results in not only physical limitations but also broad autonomic impairment affecting almost all physiological systems, and contributes to increased risk of mortality (1). Cervical SCI is the most common level of injury, accounting for over 50% of SCI cases (2). Cardiovascular complications arising from autonomic dysfunction are among the leading causes of mortality and morbidity in individuals with high-level SCI (at or above T6). These complications often coexist with other significant issues, including bladder and bowel dysfunction, sexual dysfunction, visual changes, and hormonal and immunological deficiencies (3–5).

The cardiovascular complications include arrhythmias, low resting blood pressure (BP) and significant BP lability due to poor modulation of sympathetic and parasympathetic tone (6, 7). Additionally, some individuals experience a drastic fall in BP on shifting from supine to an upright position, called orthostatic/postural hypotension (OH). This condition is exacerbated by inefficient short-term BP regulation, which stems from impaired baroreceptor reflex mechanisms (7). These reflexes are compromised due to interruptions in sympathetic excitatory pathways from the vasomotor center of the brainstem (8). Although OH is frequent and more bothersome during the early stages following high-level SCI (9), it can also manifest in the chronic phase, especially with moderate and severe cervical injuries (10). Symptoms of OH may severely affect cognitive functions and mental states with a range of associated symptoms, which severely impede rehabilitation procedures to improve functional outcomes (11–13). Further, OH increases the risk of stroke by 3–4 times after SCI (10, 14, 15).

Despite pre-existing low BP, over 90% of individuals with chronic high SCI (injury at or above T6) may experience another form of life-threatening cardiovascular dysfunction with episodic rise in BP called autonomic dysreflexia (AD) (16). In cohort of 30 individuals with high-level SCI, it was found that AD could occur as often as 41 times each day (mean = 11/day) (17). BP can rise as high as up to 300 mmHg (18). Failure to promptly diagnose and provide rapid and appropriate treatment in severe cases can lead to serious complications such as hypertensive encephalopathy, stroke, cardiac arrest, non-blockage-related cardiac ischemia, seizures, pulmonary oedema, and even death (5). Thus, it is crucial to monitor unstable BP and HR in people with high-level SCI proactively rather than waiting for symptoms to manifest.

Patients with tetraplegia consistently prioritize addressing autonomic issues, especially the elimination of autonomic dysreflexia (19). However, despite this condition and cardiovascular dysfunction in general being often poorly understood in clinical practice, with complex and challenging clinical management issues, autonomic dysfunction has been under-investigated (19). Within the last decade, alongside the assessment of motor and sensory deficits using the International Standards for the Neurological Classification of Spinal Cord Injury (ISNCSCI), the International Standards to document Autonomic Function following SCI (ISAFSCI) were developed for clinical evaluation and management of autonomic dysfunction following SCI. These were updated in 2017 and 2021 (20, 21), and further adaptations might be expected in future to refine their use in monitoring, measurement and management of autonomic dysfunction. Given that cardiovascular disorders rank among the leading causes of mortality, following respiratory and septicemia complications, in individuals with SCI during both the acute and chronic stages (22, 23), it is imperative to further investigate and elucidate the cardiovascular consequences associated with this condition.

A number of studies, focused on developing restorative therapies and pharmacological treatments, have demonstrated greater recovery of sensorimotor functions through various treatment options, including assistive technologies, rehabilitation, and reconstructive surgery (24–27). There is growing evidence suggesting that spinal cord neuromodulation could potentially facilitate motor (28, 29) and autonomic (30–33) recovery following SCI. While there has been a significant increase in the number of investigations of autonomic dysfunction after SCI in recent years, therapeutic interventions to treat cardiovascular autonomic dysfunction remain limited, with inconsistencies in methodology and outcome measures across studies. This limitation underscores the necessity for a scoping review of the measurement and management of autonomic dysfunction specifically affecting the cardiovascular system after SCI.

Therefore, the aim of this scoping review was to explore the existing literature to identify gaps in the measurement, monitoring, and evaluation of cardiovascular autonomic dysfunction after SCI, and to further explore the effects of different therapeutic interventions.

## Review questions

1. How is autonomic function, especially cardiovascular components, measured and monitored in individuals with different severity of SCI?
2. What types of treatment have been employed to manage autonomic dysfunction in SCI?

## Objectives

To provide a structured overview of the available clinical data regarding different approaches of measurement and management of cardiovascular autonomic dysfunction after SCI and to identify the research gaps in these areas.

## Methods

The scoping review adopted the methodological framework of Arksey and O’Malley (34) and followed the recent application of the framework (35–37) along with guidance for conducting systematic scoping reviews developed by Peters *et al.* (38). The Preferred Reporting Items for Systematic Reviews and Meta-Analyses extensions for Scoping Reviews (PRISMA-ScR) checklist was used (39).

The protocol for this review was pre-registered on the Open Science Framework (OSF) Registry. Registration DOI: https://doi.org/10.17605/OSF.IO/KVHQY.

## Literature search

A literature search strategy was developed for relevant databases (Ovid MEDLINE, PubMed, CINAHL, EMBASE, All Cochrane and SportDiscuss) in consultation with a reference-librarian. Additionally, relevant articles were identified by searching through reference lists and consulting authors known for their expertise in the field, as well as searching through gray literature. The search strategy contained three constructs: SCI, autonomic function parameters, and management. Autonomic keywords, particularly those describing the cardiovascular system, were deliberately kept broad to encompass the diverse methods of measuring and managing cardiovascular function. The final search terms and combinations are detailed in Appendix A. The keywords and search strategy were initially defined for Ovid MEDLINE and then replicated across all databases using controlled and free-text (title and abstract) search terms, limited to research conducted in humans and publications in English. No restrictions were placed on publication dates. The search results were transferred to Covidence (Veritas Health Innovation, Melbourne, Australia) to remove duplicates and for further processing.

## Study selection

**Figure 1:**
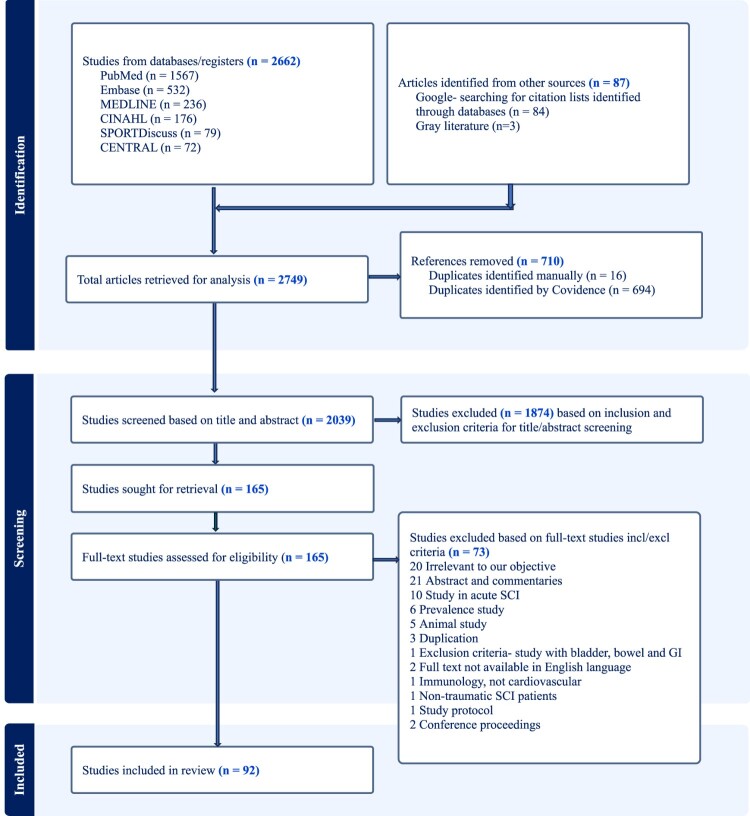
PRISMA flow diagram.

Eligibility criteria were established for both title/abstract and full-text screening (Table 1). After removal of duplicates using Covidence, titles and abstracts were screened by two independent reviewers for assessment against the inclusion/exclusion criteria. Prior to commencing the full-text assessment, the eligibility criteria were reviewed and refined by the study team. Following this, full texts were examined, and if any uncertainties arose, they were resolved by consensus. The full text of selected citations was assessed in detail by at least two authors. Any dispute arising among the reviewers during each phase of the selection process were addressed through discussion or by involving an additional reviewer. Following the completed full-text evaluation, an additional manual search was conducted to ensure comprehensive inclusion of relevant studies. The database findings, screening and references were organized with EndNote (Clarivate, Philadelphia PA). The results of the search and the study inclusion process were presented in a PRISMA-ScR flow diagram (Fig. 1). Results were grouped according to specific categories to identify the research gaps.

**Table 1:** Eligibility criteria for title/abstract and full-text screening of studies.

A. Title/abstract screening
*Inclusion criteria*
Studies of traumatic SCI in humans Interventions, such as: pharmacological, spinal stimulation, exercise, rehabilitation. Study designs including randomized controlled trials, non-randomized controlled trials, before and after studies and interrupted time-series studies, analytical observational studies including prospective and retrospective cohort studies, case-control studies, and analytical cross-sectional studies. Descriptive observational study designs including case series, individual case reports and descriptive cross-sectional studies. Relevant systematic reviews that meet the inclusion criteria, depending on the research question. Gray literature, text, opinion, relevant guidelines, papers. No limitations based on the outcomes addressed. Studies published in English language.
*Exclusion criteria*
Studies in pediatric SCI. Animal studies: our study is concerned only with human participants. Descriptions of protocols, proposed research, conference proceedings, abstracts and commentaries. Acute management in SCI. Autonomic functions other than the cardiovascular system, such as bladder, bowel and sexual functions in patients with SCI.
B. **Full-text screening**
*Inclusion criteria*
Study includes measurement of cardiovascular autonomic outcomes. Study includes management of altered cardiovascular autonomic parameters following traumatic SCI. Study investigates the prevention of autonomic dysfunctions in SCI.
*Exclusion criteria*
Study includes measure of functioning as predictor variable only. Study with outcome assessed/evaluated within the acute setting.

## Data extraction and charting of results

The data extracted from each included article were tabulated according to the main theme addressed by the article. Methodological quality was assessed using the Critical Appraisal Skills Programme (CASP) checklist for systematic reviews and original research studies (40–42). Descriptive statistics were utilized to analyze the frequency of categories extracted from the data. Results were examined to determine trends in monitoring and measuring cardiovascular autonomic conditions over time. Additionally, different interventional components, characteristics and outcomes, as well as gaps in research were analyzed.

## Results

### Articles retrieved

The flowchart for studies retrieved is presented in Fig. 1. The initial search of the six databases yielded 2662 articles plus an additional 84 identified via an independent targeted search using the reference lists of significant recent review articles and via google search for well-known principal investigators. Three additional gray literature sources with recommendations for the management for cardiovascular autonomic dysfunction following SCI were included from national and international care providers. Therefore, a total of 2749 articles were retrieved to begin the analysis using Covidence. Among them, 710 duplicate articles were excluded; Covidence identified 694, and an additional 16 were identified manually. The remaining 2039 studies were screened based on inclusion and exclusion criteria for title and abstract (Table 1(A)). In the title and abstract screening process, 1874 studies were excluded, leaving 165 studies for full-text review. These were assessed against the inclusion and exclusion criteria for screening full text (Table 1(B)). Of 165 articles, 73 were excluded (Fig. 1) because they did not meet eligibility criteria. Finally, 92 studies were included in this review.

### Study sample and characteristics

**Figure 2:**
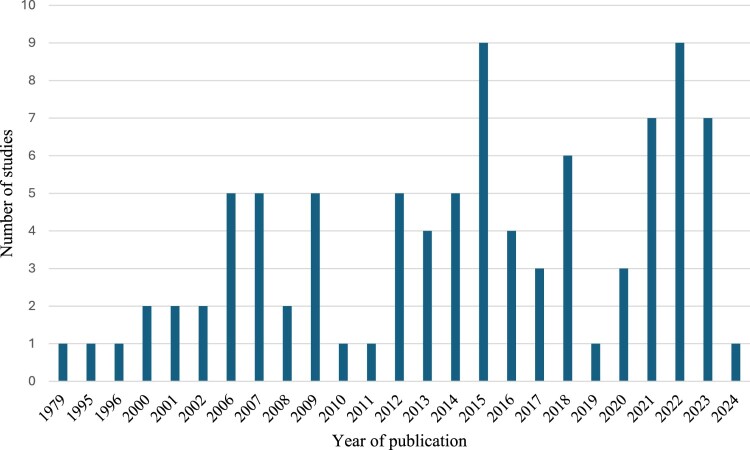
Bar chart depicting the number of studies included in this review of the measurement and management of cardiovascular autonomic outcomes (*N* = 92).

**Figure 3:**
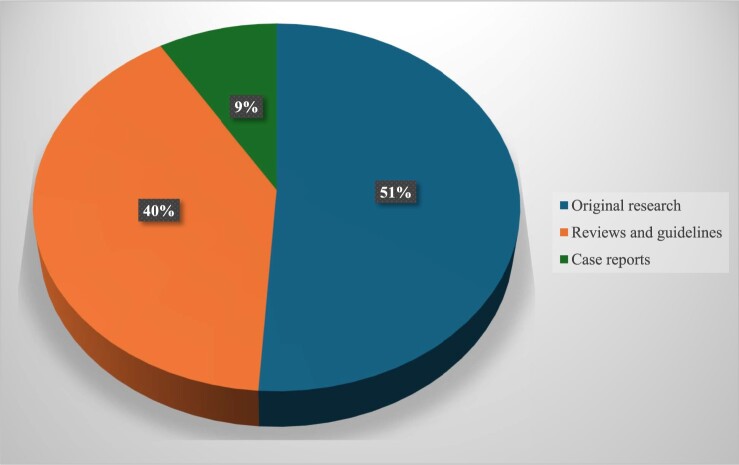
Distribution of different types of studies included in this review (*N* = 92).

While the earliest article identified was published in 1979, the frequency of studies related to measurement and management of cardiovascular autonomic dysfunction markedly increased in the last decade (Fig. 2). Of the 92 studies, 37 (40%) articles were review articles and guidelines, including 10 systematic reviews, 21 narrative reviews, and six guidelines. Forty-seven (51%) were original research articles, and the remaining eight (9%) were case studies (Fig. 3). Study quality was variable.

The articles reviewed were grouped according to whether they were narrative reviews focusing on the pathophysiology and clinical manifestations of cardiovascular autonomic dysfunction (Table 2), measurement (Table 3) of autonomic symptoms, reviews focusing on the management (Table 4) or guidelines regarding the documentation and management of autonomic dysfunction (Table 5), original research articles, including case studies, concerned with measurement and management of autonomic dysfunction (Table 6), and systematic or scoping reviews of interventions to manage autonomic symptoms or to attenuate cardiovascular risk (Table 7). Detailed summaries of these articles are documented in the respective Tables, organized according to date.

**Table 2:** Pathophysiology and clinical consequences of cardiovascular autonomic dysfunction after traumatic SCI.

Author, year, country	Study design	Topic	Outcomes and Conclusions
Teasell *et al*. (43), 2000 (Canada)	Narrative review	Pathophysiology of clinical consequences of disruption of supraspinal sympathetic nervous system control after SCI	*Pathophysiology*: - *Reduced sympathetic activity*: sparse activity in cutaneous and muscle postganglionic axons during control conditions and during bladder stimulation; reduced catecholamine and dopamine hydroxylase levels in cervical SCI - *Morphologic changes in sympathetic preganglionic neurons*: atrophy immediately after injury then regaining of normal morphology; in one case, significant decrease in soma area of neurons in the intermediolateral cell column below injury *Peripheral alpha-adrenoceptor hyperresponsiveness*: enhanced pressor repose to noradrenaline after Cx SCI; *Clinical cardiovascular consequences:* - *Low BP in tetraplegia - OH*: pooling of blood in the viscera and lower limbs; improves over time - reasons unclear but could include vascular wall receptor hypersensitivity, increased skeletal muscle tone, some recovery of postural reflexes at a spinal level, adaptation of the renin-angiotensin system. - *Loss of diurnal variation in BP and plasma noradrenaline levels* - *Bradycardia*: persistent bradycardia in severe Cx SCI; tracheal stimulation may lead to cardiac arrest due to unopposed vagal overactivity. - *Cardiovascular response to exercise*: Cx SCI negatively affects cardiac performance and maximal cardiac output remains low after exercise; some WC athletes intentionally increase their BP or induce hyperreflexia to improve performance. - *AD*: develops over time and therefore may be related to peripheral and/or central receptor hypersensitization; Treatment with prazosin (alpha-adrenoreceptor antagonist) led to fewer episodes of AD. - *Pressure ulcers*: arteriovenous shunting could increase susceptibility to skin ulceration, as does pressure-induced skin blood flow occlusion; could be related to alpha-adrenoreceptor hyper-responsiveness
Claydon *et al*. (44), 2006 (Canada)	Narrative review	Pathophysiological basis for the development of orthostatic hypotension after SCI.	Orthostatic hypotension (OH) common in acute SCI but persists in chronic SCI; experienced during orthostatic maneuvers, physiotherapy and mobilization. - More common in tetraplegia (higher SCI). - Elderly people with SCI experience relatively less OH, reason unknown. - Precise mechanism of OH not defined clearly, likely multifactorial- Sympathetic nervous system dysfunction, altered baroreceptor sensitivity, lack of skeletal muscle pumps, cardiovascular deconditioning, altered salt and water balance. - Parasympathetic control is usually preserved in SCI but the synergistic relationship between parasympathetic and sympathetic control is lost, particularly in those with Cx and HT SCI. - Disruption of spinal sympathetic pathways affect the vascular resistance responses to orthostasis, particularly in the dependent parts of the body. - Although circulating catecholamine levels, particularly noradrenaline, in supine are low in high SCI, there is a coincident hyper-responsiveness of alpha adrenoceptors below the lesion site. - Noradrenaline levels are abnormally low while supine, and there is failure to increase catecholamine levels in response to an orthostatic challenge. Management of OH involves advice and avoidance of precipitating factors, sleeping with bed head raised by 10–20°, increased salt and fluid intake, abdominal binder and/or support stockings.
Krassioukov and Claydon (7), 2006 (Canada)	Narrative review	Pathophysiology of cardiovascular dysfunction after spinal cord injury- based on clinical observations and animal model.	Pathophysiology involves disruption and plastic changes in sympathoexcitatory pathways, sympathetic preganglionic neurons, spinal interneurons, afferents, peripheral neurovascular components, all of which cause autonomic instability. *Autonomic dysreflexia (AD)*: - Life-long problem, more prevalent in chronic phase, in tetraplegics (T8 or above), can be symptomatic or asymptomatic; triggered by noxious and unpleasant stimuli; can have serious consequences even death. - Management of AD: identify trigger, adjust posture to upright position, loosening of tight clothing, monitor BP every 5 min, use antihypertensive (if indicated). *Orthostatic hypotension (OH)*: - Multifactorial mechanisms; secondary to a reduction in sympathetic activity below injury; common in the acute phase of injury. - Management of OH: recognition of the symptoms, change posture to a horizontal position and elevate the lower extremities to prevent cerebral ischemia; plasma expansion and avoidance of excessive venous pooling (extremity compression and drugs)
Krassioukov (4), 2009 (Canada)	Narrative review	Pathophysiology and management of autonomic dysfunctions related to cardiovascular (AD and orthostatic hypotension) and respiratory functions following cervical SCI	*AD:* - In general, resting BP lower in tetraplegia (T6 or above), however, AD may be experienced daily or several times per day, and may be symptomatic or asymptomatic. - BP may exceed 300 mmHg. - AD frequent in tetraplegia with 3 times more in complete injury. - Mostly occurs in chronic stage. - Untreated AD leads serious consequences and is fatal. *Pathophysiology underlying AD:* - Loss of supraspinal input to the spinal sympathetic circuits. - Reduced overall sympathetic activity, altered of spinal reflexes, plastic changes within the spinal cord (sympathetic preganglionic neurons, interneurons, dorsal root afferents), and plastic changes within the peripheral autonomic circuits (autonomic ganglia and peripheral alpha-adrenoceptors). *OH:* - In addition to the low resting BP, further drop in BP on shifting posture; particularly common in acute cases; symptoms include fatigue or weakness, light-headedness, dizziness, blurred vision, dyspnoea, and restlessness; OH can be asymptomatic in 41.1% of cases. *Pathophysiology of OH:* - Loss of sympathoexcitatory efferent pathways from the brainstem to the spinal SPNs involved in vasoconstriction causes failure of short-term reflex blood pressure regulation; leads to a pooling of blood in the viscera and dependent vasculature below the level of injury; impaired baroreflex response. - Resting catecholamine levels are lower in individuals with cervical SCI compared to those with paraplegia and able-bodied individuals. - No significant increase in epinephrine or norepinephrine levels when tetraplegic individuals undergo a head-up tilt. *Respiratory functions:* - Loss of diaphragmatic voluntary inspiration is the most prominent feature of high cervical SCI. - Preservation of the integrity of the C3–C5 spinal segments following SCI is crucial for spontaneous breathing in individuals with SCI.
Grigorean *et al*. (45), 2009 (Romania)	Narrative review	Review of cardiac dysfunction after SCI	After acute stage of SCI, cardiac dysfunction is noted by: - Cardiac dysrhythmias (bradyarrhythmias and tachyarrhythmias) correlated with level and severity of SCI. - Cardiovascular deconditioning – prolonged inactivity, loss of orthostatic tolerance - Autonomic dysreflexia - Coronary heart disease - May be avoided, treated or decreased by proper prophylactic measures; curative treatment are limited.
Weaver *et al*. (46), 2012 (Canada)	Narrative review	Review of disordered cardiovascular control after SCI, including pathophysiology	*Neurogenic shock:* Severe hypotension and persistent bradycardia; correlated with severity and level of injury; can last for up to 5 weeks post-injury. *OH*: More severe in Cx SCI. *AD*: Occurs in 50–90% of people with Cx and high Tx SCI; variable; may be silent; HR decreases because of increased vagal activity. *Cardiac arrythmias:* - Higher incidence of repolarization in SCI. - More risk of premature atrial contractions, interventricular conduction delays and bundle branch blocks. - Incidence of arrythmias (especially bradyarrhythmias) in higher level injuries. - Incidence of cardiac arrhythmias may be under-estimated. *Temperature dysregulation:* - After high SCI there is a large area of skin blood vessels disconnected from sympathetic control. - Poikilothermia and fever common after SCI. - Elevated sacral skin temperature may be a risk factor for development of pressure ulcer. - Lack of sweating contributes to thermodysregulation. *Pathophysiology OH:* - May result from interruption of sympathoexcitatory efferent pathways from brainstem to the spinal preganglionic neurons causing failure of short-term reflex BP regulation; pooling of blood in viscera and dependent vasculature below the injury. - Lack of continuous reciprocal activation of postural skeletal muscles and respiratory muscles (known to be important in counteracting venous pooling in the upright position) leading to pooling of blood in the viscera and dependent extremities. - Plasma catecholamine levels lower in Cx SCI, consistent with inability to produce reflex increases in sympathetic nervous activity. - If head-up tilt is prolonged BP partially recovers due to activation of the renin-angiotensin-aldosterone system. *AD:* - Rapid rise in BP suggests reflex sympathetic activity and extreme cutaneous vasoconstriction. - Release of greater quantities of norepinephrine per impulse. - Reorganization of spinal pathways controlling preganglionic sympathetic neurons. - Increase in size of receptive fields of individual interneurons leading to exaggerated spinal reflex control. - Sprouting of dorsal horn CGRP afferent fibers. - Nerve growth factor (NGF) contributes to enlargement of the afferent branching in spinal cord. - Injury-induced sprouting of dorsal gray commissure neurons of the lumbosacral spinal cord (relay visceral sensory input rostrally to sympathetic preganglionic neurons). - Inflammation due to secondary tissue damage.
Hector *et al*. (47), 2013 (Denmark)	Narrative review	Review of the incidence of cardiac arrhythmias in SCI.	Individuals with chronic SCI are not at greater risk of having cardiac arrhythmia nor do they have a lower resting HR than non-injured people. - Level and severity of SCI have no influence on the prevalence of arrhythmias in the chronic stage. - AD and arrhythmia are frequently induced with electro-ejaculation *Limitations*: - Too little data to produce a well-defined cut-off level for appropriate early cardiac intervention - Lack of continuous and longitudinal data recording all arrhythmic events in individual patients from the acute period and throughout the chronic phase
Wan and Krassioukov (48), 2014 (Canada)	Narrative review	An overview of the most common documented complications associated with episodes of AD	*Life-threatening complications* - 26 studies described 32 cases of life-threatening complications or death associated with episodes of AD.; total of 7 (22%) cases where episodes of AD resulted in death; 6 due to CNS-related complications, 1 due to pulmonary oedema. *CNS-related complications:* 23 cases. - Hemorrhage most common (48%), cerebral ischemia or infarction (17%) seizures or convulsions (39%) *CV complications: 7 cases* - One resulted in cardiac arrest; five involved arrhythmias; one resulted in silent myocardial ischemia; none resulted in death *Pulmonary oedema*: 2 cases; one death.
Phillips and Krassioukov (15, 49), 2015 and 2017 (Canada)	Narrative review	Review of: - autonomic control over cardiovascular system - mechanisms underlying CV abnormalities after SCI - end-organ consequences - management of AD	Multiple potential triggers for AD; most common are bladder and bowel distension. OH contributes to an elevated risk of stroke; resting hypotension plays a role in cognitive dysfunction 6 neuroanatomical changes influence autonomic cardiovascular control: - Initial sympathetic hypoactivity due to loss of supraspinal tonic sympathetic excitation - Alterations in the morphology of SPNs - Plastic changes of the spinal circuits (*i.e.* dorsal root afferent sprouting, potential formation of aberrant synaptic connections) - Altered sympatho-sensory plasticity. - Altered peripheral neurovascular responsiveness - Cumulative effect of tertiary factors (“perfect storm” of reorganization predisposing to AD) *Changes over time Acute*: - Neurogenic shock (severe hypotension and bradycardia) *Long-term*: - Interacting secondary conditions affect development of CVD - Physical inactivity. - Impaired glycaemic control. - Inflammation. - Lipid abnormalities. *End-organ maladaptation* - Arterial dysfunction - central arterial stiffness. - Heart structure and function: reduced heart size (especially high Tx and Cx injuries). - Brain: cognitive function is impaired (due to low BP) and stroke risk in 2–3 times greater in SCI. *Potential prevention/mitigation strategies (pre-clinical)*: - Stem cell (autologous olfactory ensheathing cells) transplants to improve AD. - Reduction of inflammation (inhibition of leukocyte migration across BBB). - Reducing CGRP related sprouting in dorsal horn *Clinical management of AD* - Education on bladder, bowel and skin care. - Resolution of triggers to AD. - When these measures fail to reduce BP pharmacological interventions required: nifedipine (Ca++ channel blocker), captopril (ACE), nitropaste (vasodilator), however these may exacerbate low resting BP OH. - Appropriate fluid intake. - Avoid diuretics, large meals and heat stress. - Wearing compression stockings. - Potentially adopt a semi-upright sleeping position. - Pharmacology management may include fludrocortisone (volume expansion) and midodrine (alpha 1 agonist)
Phillips & Krassioukov (50), 2016 (Canada)	Narrative review	Review of pathophysiology of cardiovascular autonomic abnormalities following SCI and impact on exercise performance.	*Sympathetic system* - Increases HR, cardiac contractility, vascular constriction, and increases BP. - Rostral ventral lateral medulla is the major sympathetic cardiovascular area responsible for BP regulation - Sympathetic neurons from here project to spinal cord via dorsolateral funiculus to synapse on spinal sympathetic preganglionic neurons (SPN) in lateral horns of T1–L2 spinal gray matter *Parasympathetic system* - Reduces HR and contractility; does not supply systemic blood vessels with some exceptions - Parasympathetic division: cranio-sacral output; cranial nerve X (vagus nerve) supplies heart and cerebral blood vessels - Spinal segments (S2–S4) do not affect cardiovascular control *Baroreceptor reflex* - Short-term BP regulation also plays important role in long-term regulation. *Pathophysiology of cardiovascular dysregulation after SCI* (based on findings from animal (rat) model): - Morphological changes in spinal circuitry - SPN plasticity. - Dorsal root afferent and intraspinal plasticity - Vascular peripheral component *Clinical consequences after SCI*: low BP, AD, OH *Implications of AD on exercise*: - BP and HR decrease in response to exercise, leading to reduced exercise performance - Classification of athletes after SCI should not only be based on motor function but also on aerobic performance which is dependent on cardiovascular autonomic function.
Biering-Sørensen *et al*. (1), 2018 (Denmark)	Narrative review	Update of the current knowledge related to the alterations in cardiac autonomic control following SCI	*Anatomical changes:* - Disruption of descending spinal cardiovascular pathways - Initial sympathetic hypoactivity due to loss of supraspinal tonic sympathetic excitation - Plastic changes of the spinal circuits including morphology of SPNs - Altered peripheral neurovascular responsiveness *Neurogenic shock*: - SBP <100 mmHg; HR <80bpm - Bradyarrhythmias, atrioventricular conduction block and hypotension. *AD* - Symptoms due to abnormal excitation of autonomic nervous system above and below the level of injury. - Conservative management with adjusting posture, removing triggers and using antihypertensive drugs, if necessary. - HR variability parameters can be used to assess cardiac autonomic activity and status of heart rate and blood pressure in tetraplegic and paraplegic SCI groups. - Echocardiography reveals significant changes in cardiac structure and function following SCI, similar to changes seen in deconditioned people - heart atrophy, especially left ventricular mass. Changes in treatment of SCI may explain conflicting results *Recommendations* - Longitudinal studies needed. - Use International SCI Cardiovascular Basic Data Set (ISAFSCI).
Calvo-Infante *et al*. (51), 2018 (India)	Narrative review	Review of cardiovascular complications in acute and chronic patients with SCI and management	*OH:* - Much more frequent in patients with Cx or upper Tx spinal cord injuries due to the lack of sympathetic innervation on the cardiovascular system. - More severe in acute than in chronic SCI - Need to maintain an optimal state of hydration. *Dysautonomia* - Patients with high Tx (T6 or higher) or Cx trauma may experience sudden episodes of peripheral vasoconstriction accompanied by dysautonomia - Most common stimuli are intestinal or bladder distention - Management is conservative, eliminating the harmful stimulus and waiting for an adequate hemodynamic response, ACE inhibitors, calcium antagonists, alpha 1 blockers or intravenous nitrates *Coronary heart disease* - 20% of deaths from spinal cord trauma considered due to physical inactivity, obesity, hyperlipidemia, insulin resistance and diabetes. - In the chronic phase, there is usually an increase in LDL cholesterol and a decrease in HDL, the reason still unclear, but assumed to be due to lack of exercise, adrenergic dysfunction, and inappropriate diet
Eldahan and Rabchevsky (52), 2018 (USA)	Narrative review	Review of the pathophysiological mechanisms associated with the development of AD	Magnitude of hypertension required to be considered AD varies across studies. *Mechanisms contributing to AD:* - Loss of supraspinal control over sympathetic ganglionic neurons; these modulate the tonic firing of sympathetic preganglionic neurons (SPN) which, in turn, send projections to the peripheral sympathetic chain ganglia or directly to the adrenal medulla which secretes epinephrine and norepinephrine (NE) into the circulation. - Synaptic reorganization of sympathetic preganglionic neurons (SPN). - Primary afferent sprouting, including nociceptive afferents. - Propriospinal plasticity -here is compelling evidence for functional plasticity of propriospinal interneurons which comprise spinal sympathetic circuits after transection SCI in the rat. - Peripheral adrenergic hypersensitivity. *Clinical management of AD* - Immediate measures involve assessment of resting arterial pressure and monitoring for other symptoms associated with AD. *Systemic effects of recurrent AD:* - Repetitive surges in blood pressure from recurrent AD may induce maladaptive structural changes in peripheral vasculature. - Diminished basal contractility of heart. - Emerging reports of associations between AD and cerebrovascular function. - AD is linked to aberrant functioning of the immune system. Important questions remain regarding the underlying mechanisms responsible for episodic AD that develops after SCI.
Brown *et al*. (53), 2018 (Australia)	Narrative review	Review of sympathetic responses to innocuous and noxious sensory stimulation below lesion in SCI	- AD is assumed to be triggered by noxious inputs below the lesion. - However selective noxious stimulation of skin or muscle in people with SCI does not cause an increase in sympathetic vasoconstrictor drive, although the same stimuli cause strong sensations of pain in able-bodied subjects. - Conversely, increases in vasoconstrictor outflow that can lead to AD can be evoked by non-noxious stimuli in SCI (weak electrical stimulation over the abdominal wall; penile vibration; non-noxious bladder distension during urodynamics), which activate large diameter somatic afferents, - Important to recognize the role of non-painful stimuli below the spinal lesion in triggering AD.
Wulf and Tom (54), 2023 (USA)	Narrative review	Sympathetic innervation to different organ systems after SCI	Both sympathetic and parasympathetic nervous systems are modulated via descending supraspinal control from different areas of the brain *Plasticity* following SCI contributes to sympathetic dysregulation: - Nociceptive primary afferent fiber sprouting in dorsal horn and in laminae VII/X around the central canal - Propriospinal neuron and interneuron plasticity - SPN plasticity – morphological changes and loss of descending inhibition *Mechanisms* still not fully understood; could include: - Neuroimmune system – persistent activation of both central and peripheral neuroimmune and inflammatory processes - Inflammatory response – activation of microglia and other immune cells releasing cytokines (TNFα, IL-1β, IL-6) and neurotrophic factors (NGF, BDNF) - SCI-induced expression of thrombospondins (TSP), proteins known to stimulate synapse formation. *Dysfunction of organ systems following SCI:* cardiovascular system, respiratory system, liver, gall bladder, pancreas, gastrointestinal tract, spleen, adrenal gland, urinary system, reproductive system. *Cardiovascular system:* - Hypotension - Autonomic dysreflexia – intraspinal plasticity leads to sympathetic hyperexcitability, which is further intensified over time; associated with detrimental remodeling of the peripheral vasculature including upregulation of adrenergic receptor increasing the sensitivity to vasopressors.
Pavese and Kessler (55), 2023 (Italy)	Narrative review	Review of prediction of lower urinary tract, sexual, and bowel functions and AD after SCI (only AD reviewed here)	*AD* - Predictors derived from investigations of onset of AD during urodynamic studies; lesion at T6 or above and presence of neurogenic detrusor overactivity. - Prevalence of AD is two to three times higher in patients with complete injury.

**Table 3:** Measurement of autonomic dysfunction after traumatic SCI.

Author, year, country	Study design	Topic	Outcomes and Conclusions
Brown *et al*. (56), 2007 (Australia)	Cross-sectional study	Assessment of cutaneous vasoconstriction as an indicator of increases in sympathetic activity in SCI (*n* = 20, C3–T11) and 9 healthy controls - Cutaneous electrical stimulation applied to forehead and abdominal wall - Monitoring of sudomotor (GSR amplifier) and vasomotor responses (infrared pulse plethysmography), BP, HR and respiration (strain gauge transducer)	- Scarce sudomotor (electrodermal) responses to forehead stimulation in SCI participants. - Cutaneous vasoconstrictor responses (photoelectric pulse plethysmography) provided a sensitive indicator of remaining central control of sympathetic function below the lesion. - Electrical stimulation applied to the abdominal wall evoked vasoconstrictor reflexes below the lesion in the majority of SCI participants, whereas only a limited number of electrodermal responses were observed. - These cutaneous vasoconstrictor responses reflected parallel increases in muscle and splanchnic vasoconstrictor activity as indicated by increases in BP; participants lacking vasoconstrictor responses rarely showed stimulus-induced BP increases. *Conclusion:* Skin vasomotor responses to somatosensory stimulation provide a more sensitive tool than electrodermal responses for evaluation of sympathetic function in SCI.
Berger *et al*. (57), 2014 (Canada)	Narrative review	Review of SSR (sympathetic skin response) and autonomic dysfunction	- Traditional stimulation sites: supraorbital, median nerve, peroneal or tibial nerve, visual; inspiratory gasp. - Stimulus is a square wave pulse strong enough to elicit a motor response or pain. - Responses measured using active electrodes on palm of hand or plantar surface of foot, reference electrodes on dorsum of hand or foot. *SSR:* - Useful and practical in assessing integrity of autonomic pathways. - Associated with AD and OH - If used alone, may not be indicative of presence or absence of autonomic impairment - Represents only sympathetic cholinergic pathways - Factors contributing to variability include habituation, skin temperature, level of emotional arousal, age (less likely >60 years), stimulus strength - Stimuli delivered above lesion may provide more robust information - Full characterization of autonomic dysfunction requires a battery of tests including SSR, BP response to Valsalva, & perturbation below level of lesion (discordance between sympathetic cholinergic and adrenergic tests)
Hubli and Krassioukov (58), 2014 (Canada)	Narrative review	Discussion of the clinical practicability of ambulatory BP monitoring (ABPM)	ABPM is superior to clinical BP monitoring re prognosis for CV morbidity and mortality, especially in nocturnal and morning windows. *BP* - *Nocturnal BP* is the most sensitive predictor of CVD (especially if no or diminished reduction in BP) - Mean arterial BP is determined by the combination of cardiac output and total peripheral resistance - Pressure in arterial system is regulated on a beat-to-beat basis by ANS; long-term adaptations controlled by renin-angiotensin system - Diurnal oscillation in BP is under control of biological clocks and contributed to by sympathetic nervous system - In normotensive people, BP shows a peak in early morning and a trough during sleep (dipper pattern) - Physical activity is the major factor influencing BP during the day - Higher level injuries (above T6) eliminate descending autonomic control over the splanchnic vascular bed and lower limb blood vessels which are important in regulating BP - Daytime mean systolic, diastolic and arterial pressure are lower in people with tetraplegia compared with those with paraplegia and the able-bodied; night-time values do not differ in the three groups - Low daytime BP in tetraplegia is the result of (1) loss of or reduced central sympathetic drive, and (2) lower levels of daily physical activity - Loss of nocturnal dip in tetraplegia occurs because daytime BP is low (maintained in low and high thoracic injuries) - Associated with low plasma norepinephrine levels and with sudden hypertensive events (AD) - Loss of circadian rhythm in BP in AIS A, but not incomplete injuries. *HR* - Inconsistent findings regarding preservation of circadian HR rhythm after SCI. - Preserved nocturnal HR dip mediated by increased parasympathetic outflow. - Loss of HR dip has been interpreted as loss of central modulation of sympathetic activity. - BP variability greater in those who recently experienced episodes of AD. - Subsequent changes occurring below the level of SCI may contribute to increased BP fluctuations: (1) plastic changes within SPNs, interneurons (2) aberrant dorsal root afferents sprouting (3) plastic changes within the peripheral autonomic ganglia and (4) changes in a-adrenoceptors sensitivity. *Practicality of APBM*: - Frequency of measures and noises made by the equipment lead to sleep disturbances. - Using diaries to record activity is impractical. - ABPM is a useful tool to investigate altered circadian BP patterns after SCI.
Currie *et al*. (59), 2015 (Canada)	Test-retest reliability	Evaluation of the reliability of the sit-up test in SCI (C4–T11, AIS A/B, 1–17 years post-injury); (*n* = 5 M, 3 F) Measures: Δ SBP and Δ DBP	- Two testing sessions 1–3 days apart. - ICC for ΔSBP was 0.79 (P = 0.006; 95% CI 0.250–0.953) - ΔDBP showed almost perfect reliability – ICC 0.92 (P < 0.001; 95% CI 0.645–0.983). - The smallest detectable differences in ΔSBP and ΔDBP were 7 and 6 mmHg, respectively. *Conclusion*: BP responses to the sit-up test are reliable in individuals with SCI.
Davidson *et al*. (60), 2017 (Canada)	Inter-rater reliability	Investigation of HR, BP and ISAFSCI assessment by two investigators on two occasions 12–14 days apart. 48 adults with chronic SCI (41 M and 72F)	Inter-rater reliability score varied from good to strong for different sections in ISAFSCI. - For General Autonomic Function, agreement was moderate to good; for Lower Urinary Tract, Bowel and Sexual Function agreement was good to strong. - Discrepancies in agreement could be due to different systems of scoring and rating (nominal or ordinal) in different components of assessment and patients’ lack of knowledge and communication regarding different autonomic components - Lack of a specified time frame for presentations of symptoms of OH and AD in ISAFSCI lead to variation in response by patients at different point of assessment.
El Kotob *et al*. (61), 2018 (Canada)	HRV as a surrogate measure of cardiac autonomic dysfunction	Secondary analysis of data from 56 people with chronic traumatic SCI (44 M, 12 F). HRV measures at rest: - LF:HF, LF, HF, RMSSD, pNN50	- No significant HRV differences in time and frequency domains across NLI and AIS subgroups. - LF and HF indices were positively correlated in the entire sample (*r* = 0.708, P < .0001) and among impairment subgroups *Limitations:* - Inadequate sample size; limited generalizability - LF:HF ratio inappropriate for assessment of cardiac autonomic status at rest in chronic traumatic SCI. - SSR may be more suitable
Berger *et al*. (62), 2022 (Canada)	Test-retest reliability	Examination of test-retest reliability of Valsalva maneuver (VM); Adults with traumatic SCI C3–C8 (13 males and 1 female) Measures: - Beat-to-beat BP (finger cuff photoplethysmography) - Baroreflex sensitivity (Valsalva ratio, pressure recovery time, vagal baroreflex sensitivity, total recovery).	*Valsalva ratio* (VR) and *Total Recovery* (TR) demonstrated highest reliability - *Valsalva ratio* (measure of cardiovagal function during VM) is calculated as the maximum HR generated during VM divided by the minimum HR occurring in the 30 s following the release of the VM - *Total recovery* represents the relative change in SBP from baseline to the end of Phase II of the VM *Pressure recovery time* (PRT) and *alternate adrenergic baroreflex sensitivity* (BRSa1) both demonstrated moderate reliability. - *Pressure recovery time* is a measure of sympathetic adrenergic mediated normalization of SBP. - *Adrenergic baroreflex sensitivity* (BRSa) and alternate BRSa (BRSa1) are calculated from the sympathetic adrenergic components of the BP trace (phase IIL and phase IV overshoot respectively). *Cardiovagal baroreflex sensitivity* (BRSv) was not reliable. - BRSv is measured by the slope of regression of the R-R interval in milliseconds over SBP during early phase II. Twelve of the 14 participants demonstrated reproducible VM patterns: - M pattern defined by the normal sympathetic adrenergic mediated increments in SBP during phase IIL and phase IV of VM trace. - V pattern defined by impairment or absence of sympathetic adrenergic mediated phases IIL IV of VM trace. - N pattern defined by a prolonged overshoot of baseline SBP during phase IV, possibly secondary to heightened adrenergic activity below the level of SCI. *Limitations***:** Small sample size; high SD indicating varying levels of sympathetic drive; ability to sustain 40 mmHg during VM expiration was variable; effect of medications on autonomic function was not investigated.
Kurban *et al*. (63), 2023 (Canada)	Construct validity	Assessment of the construct validity of ISAFSCI	49 participants with SCI (42 M; 37 with NLI above T6); mean age 45 ± 12 years. Measures included: - ISAFSCI - Autonomic Symptom Profile - Quality of Life of Spinal Cord Injury Patients (Qualiveen) questionnaire - Neurogenic Bowel Dysfunction Score (NBD) - International Index of Erectile Function (IIEF) - Female Sexual Function Index (FSFI) *General Autonomic Function* - Item on orthostatic hypotension under autonomic control of blood pressure section significantly correlated with the ASP measure (Orthostatic Intolerance domain question) - Bradycardia item of autonomic control of the heart section not associated with AIS and injury level T5 - Autonomic control of blood pressure item was *significantly associated* with AIS and injury level T5 - Normal control of sweating items from the autonomic control of the sweating section was *not* associated with injury level L2 - Hypothermia item under the temperature regulations section *correlated significantly* with AIS A/B and injury level above T1 - Item on impaired voluntary breathing requiring partial ventilatory support under autonomic and somatic control of the broncho-pulmonary system section was *not* associated with AIS and neurological level *Lower urinary tract, bowel and sexual function* - Correlation between ISAFSCI composite bladder score and Qualiveen Composite score was *fair and significant* - ISAFSCI composite bowel score had a *fair and significant correlation* with NBD Total score. - ISAFSCI psychogenic genital arousal item for males had a *good and significant correlation* with IIEF Erectile Function domain score - ISAFSCI male orgasm item and IIEF Orgasmic Function domain had a *fair and significant correlation*. - Correlation between the ISAFSCI and IIEF ejaculation items was *good and highly significant* - Sample size for the ISAFSCI female sexual items was too small - ISAFSCI composite bladder score *correlated fairly well* with the ISNCSCI composite pinprick score. - Correlation between ISAFSCI composite bowel score and ISNCSCI composite pinprick score was *good and significant*. - ISAFSCI psychogenic genital arousal item for males had a *good and highly significant correlation* with ISNCSCI composite pinprick score. *Conclusion*: - Construct validity of ISAFSCI (2012 1st Edition) for the General Autonomic Function component was considered weak - Construct validity stronger for the Lower Urinary Tract, Bowel and Sexual Function component based on *a priori* hypotheses. - Motor completeness did not correlate well with autonomic completeness.

**Table 4:** Management of cardiovascular autonomic dysfunction after traumatic SCI.

Author, year, country	Study design	Topic	Outcomes and Conclusions
Cragg *et al*. (64), 2012 (Canada)	Narrative review	Review of the management of cardiovascular disease (CVD) and risk factors for individuals with SCI.	Chronic SCI: - Dyslipidemia often present - Low LDL and high HDL and C-reactive protein (primarily in <40 yr olds) - Abnormal glycemic control, BP irregularities. *Recommendations:* - Regular assessment of lipid profile, high-sensitivity serum C-reactive protein (hs-CRP) and blood sugar after SCI.
Burns and Solinsky (65), 2022 (USA)	Mini review	Spinal stimulation targeting cardiovascular dysregulation	*OH* - 6 studies of epidural stimulation (electrodes between T10 and S2) and one study of transcutaneous stimulation (electrodes at T10); small sample sizes – between one and five people with SCI. - All studies resulted in increased BP and positive changes in other cardiovascular parameters when measured *AD* - 2 studies in humans - Transcutaneous spinal cord stimulation: 1 human; electrodes between T7 and T8; (prevention of AD in response to colonic distension) - Epidural stimulation: 5 humans; electrodes between T12 and L3; stimulation applied daily every 2–3 h for 18 months; AD returned if stimulation was not tapered gradually.
Mundra *et al*. (66), 2023 (India)	Narrative review	Spinal cord stimulation for motor, sensory and autonomic recovery	*Epidural and transcutaneous spinal cord stimulation* have been mainly in case studies and case series to investigate motor recovery and ambulation, with a few studies of autonomic function. A variety of stimulation parameters has been used, and functional improvements have occurred after stimulation in conjunction with functional training. - In this review, no studies of *epidural spinal cord stimulation* reported changes in cardiovascular autonomic outcomes. - Two studies of *transcutaneous spinal cord stimulation,* one case study reported increased BP & HR; and 1 case series (*n* = 6), reported improvements in HR and thermoregulation.

**Table 5:** Guidelines regarding documentation and management of autonomic function after traumatic SCI.

Author, Year, Country	Study type	Objective	Outcomes and Conclusions
Krassioukov *et al*. (67), 2007 (Canada, international panel)	Expert opinion	Development of a set of definitions and classification for disorders of autonomic function after SCI	Recommendations to recognize and assess the following: *Neurogenic shock* - Not spinal shock, which is characterized by loss of reflex function below injury - Can last for 4–6 weeks post-injury; involves severe hypotension and bradycardia - Prolonged and severe hypotension is associated with severity of SCI and can last up to 5 weeks - Operational definition: SBP <90 mm Hg in supine not resulting from low intravascular volume *e.g.* blood loss, dehydration. *Cardiac dysrhythmias* - May occur in the acute phase post-SCI but risk reduces after this phase - Evidence of late asystole requiring ventricular pacing - Higher incidence of bradyarrhythmia in tetraplegia (operational definition: decrease in HR to <60 bpm), rarely in paraplegia - Tetraplegics have higher incidence of non-specific ST segment elevation and are at increased risk of developing ECG abnormalities (premature atrial contractions, intraventricular conduction delays, and bundle-branch blocks) compared with nondisabled individuals - HRV techniques could be used to assess cardiac autonomic control *Orthostatic hypotension* - Operational definition: sustained decrease in BP >20 mmHg systolic or >10 mmHg diastolic occurring within 3 min of moving from supine to upright posture - Symptoms include dizziness, nausea, light-headedness, or faintness - Adaptation to OH results from recovery of spinal sympathetic reflexes, development of spasticity and increased muscle tone, and changes in the renin-angiotensin system *Autonomic dysreflexia* - Sudden bouts of hypertension triggered by afferent stimuli below level of lesion (especially bladder dysfunction) - Symptoms include piloerection, chills or shivering, pounding headache, paraesthesias, flushing, and sweating above the lesion level, as well as nasal congestion, anxiety, malaise, and nausea - Occurs in complete and incomplete SCI. *Temperature dysregulation* - Degree of dysregulation appears to be related to injury level and completeness of injury, but not clear - 3 types: poikilothermia (prolonged cold exposure), quad fever (fever without infectious source), exercise-induced fever (especially in tetraplegia) - Should be regularly measured. *Sweating disturbances* - Episodic hyperhidrosis associated with AD, OH or syringomyelia - Most common pattern is profuse sweating above lesion with minimal or no sweating below lesion
Alexander *et al*. (20), 2009 (international panel)	Review, expert opinions consensus	Development of a common approach for documenting autonomic function in SCI	Protocol developed to assess all autonomic functions by identifying shortcomings of ISNCSCI examination. - Validation and research-based evidence lacking - Training tools required - To be applied in conjunction with ISNCSCI
Krassioukov *et al*. (68), 2012 (international panel)	Guideline	Development of a standardized form for the documentation of autonomic dysfunction after SCI	Development of a standardized form: the International Standards to document Autonomic Function after SCI (ISAFSCI) - Measurement of HR and BP to identify bradycardia, tachycardia or orthostatic hypertension. - Some measure of respiratory function is desirable. - Patient self-report responses scored as normal (2), altered (1); absent (0) or not tested. - Use of the ISAFSCI to record autonomic dysfunction is recommended - No validation studies have been done.
Krassioukov *et al*. (69), 2020 (Canada)	Guideline	Guidance to primary care physicians regarding the management of autonomic dysfunction after SCI	- Importance of health maintenance checklist to monitor BP and avoid triggers for AD - Recognize signs and symptoms of AD and institute appropriate management procedures according to the Clinical Practice Guidelines 2001. - Recognize symptoms of OH and institute treatments to prevent or manage this: thromboembolism stockings, abdominal binder, increase water and salt intake, slow transitions from lying to sitting position, and pharmacological interventions if these are ineffective, *e.g.* vasoconstrictors (midodrine) and volume expanders (Florinef). - Recognize poikilothermia. - Hyper- or hypohydrosis may contribute to temperature dysregulation.
Krassioukov *et al*. (21), 2021 (international panel)	Clinical practice guideline	Development of clinical practice guidelines for management of BP, sweating and temperature dysregulation	- Information to guide management of AD and autonomic dysfunction is limited - Good consensus among expert panel, but most recommendations based on expert opinion and field reviews. - Evidence-based recommendation for management are limited
Wecht *et al*. (70), 2021 (international panel)	Guideline for documentation	Review of ISAFSCI	ISAFSCI should be administered in conjunction with full ISNCSCI and any time there is a change in clinical intervention affecting ANS function. - 10 min quiet rest before commencing assessment. - Examination should be done with room temperature between 20 and 25°C with 30–50% relative humidity. Patient must: - Be healthy, no current illnesses including UTI or pressure ulcer - Document all prescription medications - Refrain from caffeine, heavy meal, exertion, nicotine, cannabis, alcohol for min 4 h - Empty bladder and indicate last bowel movement - Remove all pressure garments

**Table 6:** Original research articles concerned with assessment and management of autonomic functions following SCI.

Author, year, country	Study design	CASP assess	Sample size, study population	Topic	Outcomes and Conclusions
Broecker *et al*. (71), 1979 (USA)	Retrospective observational study	Low-moderate quality	Adults with traumatic SCI above T7 and AD (6 months to 28 years post-injury); *n* = 21	Investigation of the incidence of AD during low spinal anaesthesia for urological endoscopic procedures - BP monitored pre-operatively and during the procedure	- *Low spinal anaesthesia* performed using 25 to 50 mg of 5% lidocaine in a hyperbaric solution given at the L3 to 4 or L4 to 5 interspaces. - Three other patients received epidural anaesthesia with 100 mg mepivacaine hydrochloride in the epidural space at the L2 to 3 interspace level. - No patient receiving low spinal anaesthesia suffered AD (highest SBP during the procedure was 140 mmHg and the highest DBP was 80 mmHg). - Mean SBP and DBP were 120 and 65 mmHg respectively, and no patient had an increase in blood pressure over that measured preoperatively. - No AD symptoms (sweating, headaches). No episodes of hypotension or OH occurred in the operating room or recovery room (BP <100 mmHg). - *Epidural anaesthesia* in two of three patients was ineffective: AD required treatment with ganglionic blocking agents.
Arnold *et al*. (72), 1995 (Canada)	Cross-sectional comparative study	Low-quality	6 adults with chronic SCI (C4–7) and 6 normal controls	Investigation of relationship between decreased sympathetic activity in tetraplegic patients and increased responsiveness of alpha-adrenoceptors	- Foot vein distension was measured using a lightweight electromechanical tonometer - Heart rate remained stable in both groups. - Trend toward an increase in mean arterial pressure during the infusions of noradrenaline in tetraplegic patients but not statistically significant compared with normal controls. - Vasoconstriction more pronounced in SCI patients than normal controls, which signifies the hyper-responsiveness of alpha-adrenoreceptors. - Hypersensitivity of vascular alpha-adrenoceptors may be important in the manifestation of AD in patients with tetraplegia. - Which receptor sub-type is affected more could not be concluded from this study. - Whether increased alpha-adrenoceptor responsiveness is due to increased receptor density or enhanced post-receptor mechanisms is not clear.
Clinchot and Colachis (73), 1996 (USA)	Case report		55 yr adult male, traumatic C8 tetraplegia, 6 months post-SCI	A case of autonomic hyperreflexia exacerbating the pain of reflex sympathetic dystrophy	Participant had experienced episodes of headache, sweating and hypertension for 2–3 weeks prior to admission - BP rose from 90/60 mmHg at baseline to over 190/100 mmHg during hyperreflexia, provoked by bladder distention, urinary tract infections and, occasionally, changing body posture. - There was a consistent exacerbation of left upper limb pain associated with episodes of autonomic hyperreflexia, which was remitted with resolution of the AD with a clonidine patch and low-dose nifedipine - The left upper limb pain improved with aggressive therapy concomitant with improvement in his vasomotor instability. *Conclusion:* - This case illustrates the role of the autonomic nervous system in the pathophysiology of sympathetic-mediated pain
Wang *et al*. (74), 2000 (Taiwan)	Prospective observational study	Low-moderate quality	Chronic SCI more than 6 months post-injury – 16 tetraplegia; 12 paraplegias	Assessment of alterations of autonomic nervous system function and sympathovagal balance in patients with different levels of SCI using heart rate variability analysis.	*Time domain analysis* - SDNN and SDNNi decreased in tetraplegia - No difference between groups in rMSSD and pNN50 - All night-time variables were lower in tetraplegia *Frequency domain analysis* - Power spectra of VLF, LF, HF and total power decreased in tetraplegia during both daytime and nighttime - 24-hour LF-to-HF ratio not statistically different between the two groups *Conclusions:* The findings suggest a decrease in both sympathetic and parasympathetic action in tetraplegia. This may be a consequence of adaptation to maintain sympathovagal homeostasis, and may contribute to AD.
Iellamo *et al*. (75), 2000 (Italy)	Cross-sectional comparative study	Moderate quality	Chronic SCI more than 12 months post-injury; 9 complete tetraplegia (C4–C7) and 10 normal controls	Investigation of the contribution of vagal and sympathetic mechanisms to the genesis of LF oscillations of the RR-interval. - Experiment: 10 min of supine rest, followed by 10 min of passive HUT of 70°. - All participants had continuous ECG and BP monitoring.	- Arterial pressure was less in tetraplegics in the supine position and significantly lowered on tilt, but all asymptomatic. - Resting RR-interval was longer in tetraplegic than in controls - RR-interval shortened significantly during tilt and comparable to controls - All tetraplegics had HF component, however LF component was present in only one patient in supine. - Reason for such LF oscillations in RR-interval are controversial; may be due to sympathetic modulation on cardiac pacemaker cells sympathoexcitatory neurons in the medulla, or vagal efferent activity to the heart and baroreceptor stimulation. - Baroreflex sensitivity (BRS) was comparable at rest and underwent a significant and similar decrease during tilt in both groups; accompanied in controls by a significant increase in LF relative power. - Unlikely that change in efferent vagal activity to the heart from baroreflex stimulation by spontaneous arterial pressure changes alone contribute LF oscillations in people with SCI who lack the ability to modulate sympathetic nerve traffic to the heart. - However, the possibility that a baroreflex modulation of LF oscillations requires intact sympathetic control should be carefully considered.
Hohenfellner *et al*. (76), 2001 (Germany)	Interventional study	Low-moderate quality	Traumatic SCI (mean 52.5 months post-injury); *n* = 9.	Surgical denervation of bladder for the treatment of detrusor hyperreflexia and/or AD	- In all patients both SBP and DBP reduced significantly after sacral rhizotomies and in 5 patients AD was abolished completely after surgery.
Bisharat *et al*. (77), 2002 (Israel)	Case report		80 yr old female, traumatic paraplegia, T7	A case with an unusual cause for recurrent orthostatic syncope.	- Tilt table testing revealed that the patient lost consciousness without hypotension - EEG monitoring results were consistent with cerebral hypoxia - Transcranial Doppler showed a significant reduction in middle cerebral artery diastolic blood flow velocity during head upright tilt - Treatment with β-blockers was highly effective *Conclusion:* - Cerebral blood flow dysregulation was probably due to abnormal baroreceptor responses triggered during orthostatic stress
Colachis (78), 2002 (USA)	Retrospective case report		35-year-old male with C5 tetraplegia	Report on hypothermia associated with AD	- This patient experienced excessive sweating and hypothermia associated with AD. - Thermoregulatory dysfunction may be associated with excessive sweating
Claydon *et al*. (79), 2006 (Canada)	Prospective clinical research study	Moderate quality	Chronic SCI (*n* = 19 Cx and 8 Tx)	Examination of cardiovascular responses to a brief period of exhaustive arm cycling exercise in subjects with cervical- and thoracic-level SCI.	- Resting BP & HR lower in Cx than Tx SCI (MAP: Cx 76.6 ± 2mmHg; Tx 93.5 ± 3mmHg). - Post-exercise, HR responses were greater in Tx than Cx SCI; MAP increased in thoracic SCI (8.4 ± 5mmHg) and markedly decreased in cervical SCI (−9.3 ± 2mmHg) - No significant ECG abnormalities at rest or during exercise. - Significant correlations between the number of palmar SSRs and max HR rise and BP responses to exercise *Conclusion:* Abnormal cardiovascular responses to exercise and transient post-exercise hypotension common in Cx but not Tx SCI
Claydon *et al*. (80), 2006 (Canada)	Cross-sectional comparative study	Moderate-high quality	Adults with chronic SCI; 14 Cx and 11 Tx; 18 able-bodied controls	Development of a standardized clinical test to evaluate orthostatic cardiovascular control in SCI - SSR recorded from hands and feet - Continuous recordings for ECG, beat-to-beat BP - 15 min supine rest, then Sit-up Test	*Sit-Up Test:* - Lower supine HR, SAP, and noradrenaline levels in Cx than Tx SCI and controls, and lower DAP and MAP than controls - When upright, HR increased in all groups - SAP, DAP, and MAP increased in Tx SCI and controls, but not in Cx SCI - Larger postural falls in stroke volume and cardiac output in Cx SCI, with smaller increases in TPR than the other two groups - Cardiovascular control during orthostasis was impaired and OH was common in Cx, but not Tx SCI - Upright catecholamine levels were lower in Cx than thoracic SCI and controls - Severity of SCI assessed by AIS scale did not necessarily correlate with severity of damage to descending spinal autonomic pathways assessed by SSR - SSR may identify those at greatest risk of orthostatic hypotension and impaired cardiovascular control *Recommendation* - Assessments of autonomic function should be included in the neurological evaluation of SCI, in addition to the ISNCSCI assessment *Limitations:* reproducibility of procedure and findings not verified on large scale
Krassioukov and Harkema (81), 2006 (Canada)	Controlled experimental study	Low-moderate quality	Chronic traumatic SCI, 6 Cx, 5 Tx and 9 able-bodied controls	Assessment of BP and HR changes with and without harness application during different postures: sitting with and without harness, supine with and without harness, standing with harness.	- Resting arterial BP and HR significantly lower during sitting in Cx SCI than in Tx SCI and able-bodied individuals. - Orthostatic stress significantly decreased arterial blood pressure only in individuals with cervical SCI; ameliorated by harness application - Harness application had no effect on cardiovascular parameters in able-bodied individuals - DBP was significantly increased in those with SCI with harness application. - While standing with harness, individuals with Cx SCI still developed orthostatic hypotension. *Conclusion:* - Level of injury influences baseline cardiovascular parameters - Application of a harness in individuals with SCI could alter baseline cardiovascular parameters and the response to orthostatic stress - This should be carefully considered when assessing effects of therapeutic interventions using BWS in individuals with SCI
Catz *et al*. (82), 2007 (Israel)	Controlled experimental study,	Moderate quality	Adult chronic traumatic SCI, 10 Tx (T4-T6), 11 Cx (C4–7), 13 healthy controls	Examination of the autonomic cardiovascular response to cold application to hand and foot in SCI (immersion in ice water). - Continuous monitoring of HR, BP, and respiratory chest and abdominal movements - Transcranial Doppler to compute cerebral blood flow velocity (CBFV) in middle cerebral artery.	- HR increased during hand immersion and decreased during foot immersion in both tetra- and paraplegics; HR increased in both tests in control group. - HR, BP, and cerebrovascular resistance increased, consistent with sympathetic activation, whereas HRLF, HRHF, BPLF, and CBFV did not change significantly. - In the tetraplegia group, the mechanism responsible for the increase in BP and decrease in HR during foot immersion could be due to autonomic dysreflexia caused by the noxious (cold) stimuli, with reflex bradycardia through the baroreceptor mechanism; the increase in CBFV is a plausible consequence of the BP increase. - The lack of the supraspinal control, and the abnormal functioning of the autonomic spinal circuits, could be a cause for these changes in the tetraplegia group, but cannot explain the changes in the paraplegia group.
Haisma *et al*. (83), 2007 (The Netherlands)	Multicenter longitudinal study	High-quality	212 patients with SCI admitted to specialized rehabilitation centers	Assessments performed at the start of active rehabilitation (*n* = 212), 3 months later (*n* = 143), at discharge (*n* = 191) and 1 year after discharge (*n* = 143). - Complications between each timepoint were registered on a standardized list.	- Most common complications were neurogenic or musculoskeletal pain, and spasticity, urinary tract infections and pressure sores. - However, hypotension and autonomic dysreflexia remain ongoing concerns one year post-injury, with those with complete SCI more at risk of these conditions.
Claydon and Krassioukov (84), 2008 (Canada)	Cross-sectional comparative study	Moderate-high quality	Adults with chronic SCI; 14 Cx and 11 Tx; 17 able-bodied controls	Assessment of supine and upright cardiovascular autonomic function - frequency analyses of HRV and BPV - SSR - responses simultaneously from both hands and feet - ECG - Beat-to-beat finger BP.	*HRV: Cx SCI* - Longer RRI; decreased in upright position in all groups - Markedly reduced VLF power - Reduced normalized LF power - Greater normalized HF power in upright position; significantly less in Tx group in supine - Lower LF/HF ratio in supine and upright positions *BP: Cx SCI* - Lower SAP in supine and upright positions - Lower upright DAP - Less total variance and VLF/BPV - Reduced normalized LF SAP and DAP in supine and upright positions - Higher normalized HF SAP and DAP power in supine and upright positions *Cross-spectral analysis of cardiac baroreflex control* - Supine coherence and transfer function gain similar in all groups - Lower coherence in Cx group in upright position - Decreased reflex gain in Cx group - Greater phase and baroreflex delay in Cx group *Other* - Significant correlations between supine frequency domain indices and the level of injury, integrity of descending sympathetic pathways, assessed by SSR, plasma catecholamine levels, and cardiovascular responses to orthostatic stress. - Extent of cardiovascular dysfunction and severity of injury to sympathetic pathways could be predicted using only the LF-to-HF ratio of power of RRI and LF and total power of SAP (absolute units) from the supine data. - Total power of SAP was best predictor of orthostatic hypotension
Liu *et al*. (85), 2008 (Taiwan)	Observational study	Moderate quality	People with subacute quadriplegia (within 4 months of injury); *n* = 38	Investigation of relationship between physiological responses and pre-syncope symptoms during head-up tilt. - Tilt-table testing (continuous monitoring of mean BP, SpO_2_ and ECG; LF/HF ratio). Presyncope symptoms (PS) measured using eyesight range.	- BP, HR, LF/HF, SpO_2_ (averaged over each 2-min interval), and PS were recorded at 2-min intervals. - Three measurements taken at each tilt angle: 10-min supine, and then at tilt angles of 0°, 30°, 45°, 60°, and 75° for 6 min each. - SpO_2_ and the LF/HF ratio emerged as the most sensitive indicators for detecting pre-syncope.
Yasar *et al*. (86), 2010 (Turkey)	Cross-sectional comparative study	Moderate-high quality	Chronic traumatic SCI, 19 paraplegia, 11 tetraplegia, and 27 healthy controls	Investigation of the effect of autonomic dysfunction on P-wave dispersion, as a predictor of atrial fibrillation *Autonomic function* tests: - Head up tilt (80°). - HRV with respiration (head elevated to 30°, 6 respirations per minute) - Valsalva maneuver (head elevated to 30°, hold maneuver for 15 sec) - 12-lead ECG	- 16 patients had complete SCI; 13 had lesions at T6 or above; 9 patients had autonomic dysfunction or AD - P wave duration (PWD) higher in SCI; no significant difference between complete and incomplete injuries or between paraplegia and tetraplegia - SCI below T7: greater PWD during initial sensation of vesicle filling than at rest - No significant differences between resting ECG and that recorded during urodynamics in those with SCI above T6 and those with autonomic dysfunction; those with autonomic dysfunction developed AD during urodynamics - SCI above T6 had greater PWD - No significant difference in BP between SCI and controls - Increased P-wave dispersion in patients with autonomic dysfunction shows a tendency for atrial fibrillation, a risk factor for stroke
Moerman *et al*. (87), 2011 (USA)	Retrospective chart review	Moderate quality	Traumatic Cx SCI (*n* = 106) with bradycardia, hypotension, asystole, cardiac arrest	Evaluation of the effect of a cardiac pacemaker in restoring normal heart rate	- Of the 106 patients with Cx SCI, 15 (14%) had bradycardia and 7 of those (47%) underwent cardiac pacemaker placement. - A total of 35 events occurred in 6 male patients (average age 46 years) before pacemaker placement. - Subsequent to pacemaker placement, there were zero events of cardiovascular instability - Major bradycardic episodes were reduced from 9 to 0, and incidents requiring atropine administration from 9 to 0. *Conclusion:* People with Cx SCI with life-threatening complications of bradycardia benefit from early placement of a cardiac pacemaker.
Turiel *et al*. (88), 2011 (Italy)	Pre-post study	Moderate quality	Adult patients with motor incomplete SCI due to trauma (*n* = 8; 5 Cx, 3 Tx) or spondylitic (*n* = 5) disease	Investigation of the effects of robotic-assisted treadmill training on coronary and endothelial function - 2-D echocardiography of left ventricle - LAD flow Doppler to assess coronary flow reserve - Plasma levels of asymmetric dimethylarginine (ADMA)	Statistically significant improvements in: *Coronary function* - Lower LV end-diastolic and end-systolic volumes with increased ejection fraction; increase LV interventricular septum (IVS). - Improvement of LV diastolic function (reduction of isovolumic relaxation time and deceleration time (DT) (P = 0.0405) with an increased E/A ratio) *Endothelial function* - Improvements in coronary flow reserve (CFR) - Reduced plasma ADMA levels *Inflammation* - Reduction of inflammatory status (C-reactive protein (CRP) and erythrocyte sedimentation rate (ESR)).
Sisto *et al*. (89), 2012 (Canada)	Cross-sectional study	High quality	Individuals with SCI (*N* = 350) with incomplete AIS classification C and D	Investigation of resting cardiovascular parameters and orthostatic challenge prior to participation in a locomotor training program. - Classification using the Neuromuscular Recovery Scale (NRS),	*BP and HR* - Time since injury was negatively related to resting BP while sitting. - Men had higher BP and lower HR than women. - Patients with Cx injury had the lowest BP and HR, while low Tx injury patients had the highest pressures. - High Tx injury patients had higher HR than both Cx and low Tx injury patients in all positions. - No significant relationship between time since SCI and HR. - Cx injury patients had the highest prevalence (23%) of low pressures, and low Tx injury patients had the lowest (9%). *Orthostatic challenge* - Time since SCI negatively affected systolic and diastolic BP in resting positions but not after the orthostatic challenge. *Limitations*: varying measurement times, continued medication use without control groups, and no capture of autonomic symptoms.
West *et al*. (90), 2013 (Canada)	Pre-post study - 3 experimental trials	Moderate quality	7 SCI Paralympic athletes (wheelchair rugby players) with complete motor Cx SCI	Determination of the physiological basis of exercise-induced tachycardia in athletes with motor complete SCI *Measures:* - Beat-by-beat assessment of HR and BP. - SSR - VO2 *Interventions* Trial 1: Passive sit-up tilt test (legs unsupported over the side of the bed) Trial 2: Sports-specific 4-min maximal push on a 140-m straight synthetic track Trial 3: maximal incremental arm-crank exercise ergometer	- Despite classification of AIS A or B, there was variability in resting autonomic and CV function: 4 had resting hypotension in supine; 5 had resting bradycardia. - In response to sit-up test, only 2 participants had OH; all had some preservation of SSR (ranging from minimal preservation to full preservation) - Average number of SSRs at all sites was strongly correlated with HR peak in the field, 4-min push distance, and VO2 peak; no correlation with IWRF classification and no association between average SSR preservation and IWRF. *Conclusion* This study provides strong evidence that the degree of sub-lesional sympathetic control is an important determinant of exercise performance in athletes with Cx SCI rather than increasing exercise capacity through boosting (a nociceptive stimulus, usually via restriction of urinary outflow).
Helmi *et al*. (91), 2013 (The Netherlands)	Case report		61-year-old man with acute C3/C4 SCI with post-laminectomy OH, admitted to ICU	A case demonstrating the use of inflatable external leg compression to prevent orthostatic hypotension (OH) *Measures:* - BP and HR - Peripheral perfusion index using pulse oximeter	- Systolic/diastolic BP decreased from 140/55 mmHg during supine to 60/40 mmHg during head-up tilt position, followed by pre­-syncope symptoms. - Applica­tion of a pressure-titrated inflatable ELC to the upper and lower legs was able to preserve minimum arterial pressure to prevent presyncope symptoms. - The rationale for the use of ELC is to apply external counter pressure to improve venous return and increase mean arterial BP.
Flank *et al*. (92), 2014 (Sweden)	Cross-sectional study	Moderate-high quality	Wheelchair-dependent individuals with chronic post-traumatic paraplegia; *n* = 103 M, 31 F	Investigation of relationship between self-reported moderate/vigorous physical activity and CVD risk. Measures: - Physical activity questionnaire – dichotomized sample into those with mod-vigorous physical activity or not - BP (cuff) - Blood glucose, cholesterol and triglycerides - Body weight	- One in 5 patients reported more than 30 min physical activity. *Physically active group:* - Younger than the inactive group - Lower SBP and DBP, but association between SBP and physical activity disappeared when adjusted for age. - Trend to positively influence body mass index and LDL cholesterol/HDLcholesterol ratio. ***S****ex differences:* - Men had significantly higher SBP and DBP than women, lower HDL cholesterol, higher LDL cholesterol/HDL cholesterol ratio and higher triglycerides. - No other significant differences between males and females
Evans *et al*. (93), 2014 (USA)	Retrospective cohort study	Low-moderate quality	Adults with chronic SCI with symptomatic bradycardia (*n* = 18).	Investigation of enteral albuterol for symptomatic bradycardia. - 8 patients received albuterol - 10 patients received no albuterol Chronotropic agents were used as needed for rescue treatment.	- The albuterol group had fewer symptomatic bradycardic episodes (median 1.8), compared to those who did not receive albuterol (median 4.3) - The albuterol group had significantly fewer hospital days requiring chronotropic agents compared to the non-albuterol group. - Albuterol, administered enterally, functions as a β2 adrenergic receptor agonist, increasing heart rate through direct stimulation of β2-adrenergic receptors in both the left ventricle and the right atrium. - Limitations: retrospective single-center study; timing and initial administration of albuterol were not standardized in the albuterol group.
Hubli and Krassioukov (94), 2015 (Canada)	Study of test–retest reliability	Moderate quality	Adults with traumatic SCI; C2–T11; AIS A-D; 1–383 months post-injury; *n* = 14 M, 4 F	Examination of test-retest reliability of SSR - SSR recorded in supine from hands and feet using median nerve stimulation and deep breath maneuver	- Almost perfect reliability of SSR response to median nerve stimulation (ICC 0.97 and 0.96 for hands and feet respectively). - Lower reliability to deep breath maneuver (ICC 0.89 and 0.74 respectively). - The most reliable interpretation of the SSR is by its presence or absence. - Quantitative outcomes (amplitude, frequency) are highly variable.
Zheng *et al*. (95), 2015 (Canada)	Placebo-controlled clinical trial (participants acted as own controls)	Low-moderate quality	6 chronic complete SCI patients undergoing penile vibrostimulation (PVS) who regularly experienced severe iatrogenically induced episodes of AD	Examination of the efficacy of prazosin in reducing AD severity. - Resting BP measured before and after PVS *Intervention:* 1 mg tablet of prazosin or placebo at home the night before testing (loading dose), followed by a second 1 mg tablet 2 h prior to the PVS procedure.	- All 6 participants experienced AD following ejaculation from PVS after taking either prazosin or placebo. - Significantly smaller increase in SPP after ejaculation after administration of prazosin - No difference in resting BP between prazosin and placebo trials. *Conclusion* The study provides preliminary evidence that prazosin is effective in reducing the severity of AD associated with PVS.
Currie *et al*. (96), 2015 (Canada)	Cross-sectional comparative study	Moderate-high quality	8 non-athletes and 13 athletes with chronic traumatic Cx SCI	Investigation of differences in peak heart rate (HR) and sympathetic function measures between non-athletes and athletes with chronic, motor-complete, cervical spinal cord injury (SCI) *Experimental condition*: Cycling > 50 rpm on an electrically braked arm-cycle ergometer with incremental load by 5 W/min until fatigue. *Measures:* - Brachial artery SBP and DBP - Peak HR - SSR (bilateral)	- First study to demonstrate that attenuated peak HR in non-athletic individuals with tetraplegia may be secondary to impaired descending sympathetic control, as evidenced by the presence of OH and the absence of palmar SSR. - Differences were observed in peak HR and sympathetic function between non-athletic and athletic individuals with tetraplegia, despite sharing a similar lesion level and motor­ completeness of injury. - There may be a predisposition for tetraplegic individuals with preserved sympathetic function to engage in high- performance sports and highlights the necessity to include autonomic assessments in sporting classification. - Presently, sporting classifications is a sport-specific process that considers motor, visual, and intellectual impairments. The range of exercise HR capacities within a sporting classification is likely to influence exercise performance. *Limitation*: Inability to perform the same exercise tests in both groups.
Hübli *et al*. (17), 2015 (Canada)	Psychometric investigation of the ADFSCI questionnaire	Moderate quality	30 people with SCI (C3–L1, AIS A–C; time since injury: 1 month to 30 years)	1. Correlation of the ADFSCI questionnaire with objective 24-hour ambulatory BP monitoring (ABPM) 2. Assessment of the test–retest reliability of the ADFSCI questionnaire *Measures* - 24-hr ambulatory BP monitoring - ADFSCI - Diary of activities that resulted in hypo- or hypertensive events	- People with self-reported AD had significantly higher SBP coefficient of variation and more AD events - The number of AD events over the 24-hour period and BP variability were significantly correlated with self-reported total AD score and daily AD frequency - No significant correlations between number of hypotensive events over 24-hour period and self-reported frequency and severity in ADFSCI questionnaire
Goh *et al*. (97), 2015 (Australia)	Retrospective review study	Moderate-high quality	Subacute and chronic traumatic SCI; 44 tetraplegia, 10 paraplegics referred to a specialist service.	Examination of diurnal BP patterns and nocturnal hypertension, and diurnal urine production in SCI patients with clinically significant disorders of BP control. *Measures:* - 24-hour ambulatory BP monitoring - 3-day urine volumes	- Patients were referred for orthostatic intolerance (*n* = 37), autonomic dysreflexia (*n* = 6), nocturnal polyuria (*n* = 4), elevated BP (*n* = 1) and peripheral oedema (*n* = 1). - Average BP was 111.1 ± 1.4/65.0 ± 1.2 mmHg. – only 4 patients (7%) had the normal dipping pattern of night-time BP - In 56% of patients (*n* = 30), BP at night was higher than during the day and another 37% (*n* = 20) were non-dippers. - Nocturnal hypertension was present in 31% (*n* = 17) of the patients. - In the tetraplegic patients, urine flow rate was greater during the night than day - Ambulatory monitoring revealed a substantial prevalence of reversed dipping or nocturnal hypertension, despite a relatively low prevalence of 24-h or daytime hypertension; these abnormalities tended to be more frequent amongst patients with acute, complete or tetraplegic injuries. - In addition to the higher incidence of reversed dipping of BP, there was higher nocturnal urine production in patients with acute or complete SCI when compared with patients with chronic or incomplete injuries, respectively.
Aslan *et al*. (98), 2016 (Canada)	Case-control study	Low-moderate quality	11 adults with chronic SCI and OH.	Investigation of the effect of pressure threshold inspiratory-expiratory respiratory motor training (RMT) on OH. *Measures:* - Orthostatic stress test (with continuous recordings of blood pressure, heart rate, and respiratory rate). - Pulmonary Function tests	- Abolition of OH in 7 of 11 cases after RMT without significant fall in SBP and DBP, which immediately stabilized. - Significant improvement in forced vital capacity, low-frequency component of power spectral density of BP and HR oscillations, baroreﬂex effectiveness, and cross-correlations between blood pressure, HR, and respiratory rate during the orthostatic challenge *Conclusion*: Findings indicate increased sympathetic activation and baroreﬂex effectiveness in association with improved respiratory-cardiovascular interactions in response to the sudden decrease in blood pressure. *Limitation:* - Small sample size - Severity of injury not assessed – this could affect outcomes of RMT
Fougere *et al*. (99), 2016 (Canada)	Prospective cross-sectional study	Moderate quality	Adults with traumatic SCI at or above T6 (*n* = 17), confirmed AD during urodynamic studies, confirmed neurogenic detrusor overactivity	Investigation of the efficacy of intradetrusor-injected onabotulinum toxin A (Botox) on reducing the severity and frequency of bladder-related AD events. *Measures:* - Urodynamic assessment to confirm the presence of AD - 24 h ABPM - AD Health-Related Quality of Life Questionnaire and the Incontinence Quality of Life Questionnaire questionnaires.	- Following the Botox treatment, 59% of the sample no longer experienced AD during the urodynamic assessment. - The remaining 41% experienced a reduction in AD severity. - 24-hr ABPM showed that the severity of AD during bladder-related events was significantly reduced following Botox treatment (reduction in both the maximum SBP and DBP during bladder-related events). *Limitations:* - The average efficacy of intravesical injections of Botox is typically 9 months - The duration of efficacy of Botox on reducing bladder-related AD frequency and severity beyond 1 month is not known.
Machac *et al*. (100), 2016 (Czech Republic)	Case-control study	Moderate quality	Adults with Cx SCI (*n* = 20); Able-bodied males (*n* = 27)	Comparison of cardiovascular responses to peak voluntary exercise on arm crank ergometer in individuals with tetraplegia and able-bodied participants. Measures: - BP response one minute after peak exercise - Peak HR - Peak VO2	- No increase in BP response to exercise in tetraplegic group. - Significantly increased post-exercise SBP post maximal exercise test and the tetraplegic group’s workload protocol. - VO2 peak in tetraplegic group was 59% and the HR peak was 73% of those of the control group’s responses respectively. - Three tetraplegic participants were at risk of severe hypotension post-exercise.
Phillips *et al*. (101), 2017 (Canada)	Pre-post interventional study	Moderate quality	4 adults with Cx motor complete SCI (C5–C6); 1 adult with Tx SCI (T2)	Investigation of the capacity of transcutaneous spinal cord electrical stimulation (TSCS) (applied between the T7–T8 spinous processes) to manage orthostatic hypotension during an orthostatic challenge. *Measures:* - Beat-to-beat BP, stroke volume and cardiac contractility - Beat-to-beat blood flow velocity in middle and posterior cerebral arteries - Participant ranking of nausea/dizziness each minute on a 10-point scale - EMG of lower limb skeletal muscles	- Orthostatic hypotension was abolished with TSCS. - Normalization of BP, cardiac contractility, cerebral blood flow, and all symptoms abrogated. - Absence of skeletal muscle contraction meant that the result was not caused by muscle pump activity - HR did not decrease with stimulation; stroke volume still reduced *Potential mechanisms:* Excitation of propriospinal and sympathetic preganglionic neurons either directly through the stimulation reaching the spinal cord or by preferential excitation of large diameter sensory axons.
West *et al*. (102), 2018 (USA)	Case report	-	Male with chronic C5 SCI, AIS B	Investigation of whether lumbosacral epidural stimulation could be optimized to control cardiovascular functions in the short term. *Measures:* - Beat-by-beat BP - Transthoracic echocardiography - Cerebral blood flow - Trunk/lower limb EMG	*Epidural stimulation:* - Resolved OH; rise in BP was well-controlled and did not lead to AD - Prevented the orthostatic induced 30% decrease in middle cerebral artery blood flow, improved neurovascular coupling, and resolved self-reported light-headedness, dizziness and poor concentration - Prevented reduction in cardiac filling during tilt - Lack of lower limb EMG showed that venous pump through skeletal muscle contraction did not cause rise in BP - May specifically modulate cardiovascular function by increasing the resting membrane potential of sympathetic circuitry via the stimulation of dorsal afferent relays.
Darrow *et al*. (103), 2019 (USA)	Case series	–	Participant 1 - female, 52 yrs, T8 AIS A, 10 years post-SCI; Participant 2 - female, 48 yrs, T4 AIS A, 5 years post SCI	Evaluation of the effects of various stimulation parameter settings on volitional movement and cardiovascular function. *Measures:* - 24-hour ABPM - Orthostatic challenge testing - Stroke volume and cardiac contractility - Blood velocity measure in left or right middle cerebral artery - Cognitive function measures - Neurogenic Bowel Dysfunction Score and Neurogenic Bladder Symptom Score	Neuromodulation (epidural spinal cord stimulation, eSCS) using implanted stimulator at approximately the vertebral T12 level; stimulation for autonomic function (CV Stim) used an electrode configuration that maximized the current density above the electrode to excite caudal preganglionic neurons (between T1 and L2). *eSCS*: - Significantly improved cardiovascular function in Participant 2 during tilt-table testing with corresponding improvements in cerebral blood flow and cognitive performance; result highly dependent on direction of stimulation (rostrally directed current) - Participant 2 regained in bowel and bladder synergy and volitional urination and reduced the time needed for bowel routine - Resulted in restoration of sexual function in Participant 2. (NB Participant 2 was younger and was 5 years post-injury) *Conclusions:* Volitional movement and autonomic function are not optimized through the same stimulator settings and configuration. Multisite stimulation (rostral and caudal) or broader coverage in general may be necessary for restoration of both volitional movement and autonomic function.
Hill *et al*. (104), 2020 (Sweden)	Cohort study	Moderate quality	Chronic traumatic SCI, C1–T6 (*n* = 25).	The Swedish Spinal Cord Injury Study on Cardiopulmonary and Autonomic Impairment (SPICA) *Autonomic Function Tests* - HR at rest in supine - BP (supine and sitting) - Questionnaires: ADFSCI, HADS, Sense of Coherence Scale, SCIM III, SCI-SET (spasticity) - ABPM and 24-hr HR	- AD and symptoms of hypotension were frequently reported (68% and 76%, respectively) indicating cardiovascular autonomic impairment. - One participant had experienced a myocardial infarction caused by AD after indwelling urinary catheter blockage. - Risk for CVD in people with severe high-level SCI is a major clinical concern. - Comorbidities of cardiopulmonary relevance implicate several factors contributing to an increased risk for the development of CVD in middle-aged people with severe high-level SCI.
Krogh *et al*. (105), 2020 (Denmark)	Case report		23-year-old male with traumatic, cervical (C6), motor-complete (AIS: B) SCI with history of AD	Eight sessions of blood flow-restricted exercise (BFRE) for the upper extremities for 4 weeks. -BP and HR and perceptual pain responses recorded repeatedly during exercise.	- During BFRE, four instances of AD were identified, including one with symptoms. - Only two occurrences of AD detected during a standard control session of resistance training without blood flow restriction. *Conclusion*: Uncertain whether different BFRE protocols can be utilized for rehabilitation in SCI patients with a history of AD without triggering further instances of AD.
Sachdeva *et al*. (106), 2021 (Canada)	Clinical case report		Chronic motor-complete Cx SCI (*n* = 1).	Single case study of effects of TSCS on AD in a person with chronic Cx SCI *Measures:* Beat-by-beat BP using finger photoplethysmography.	*Single case study:* Digital anorectal stimulation (DARS) induces AD in people with tetraplegia. *Acute prevention of AD*: - TSCS applied prior to DARS resulted in an 82% reduced rise in SBP, a 65% reduced rise in DPB and a 68% lesser reduction in HR. *Acute interruption of AD* - In ongoing DARS with induced AD, application of TCS led to a 49% rise in SBP and a 56% reduction in DBP, with no change in HR reduced rise in BP and corrected HR. - TSCS reduced the level of tonic pelvic floor muscle activity during DARS. - Temperature rise under the cathode was 1.3 °C
Dorton *et al*. (107), 2021 (The Netherlands)	Cross-sectional study	Moderate quality	Chronic traumatic SCI (≥10 years)	1. Identification of markers of obesity, injury characteristics and autonomic function variables related to CVD risk after SCI (SCI). 2. Establishment of cut-off points for detection and risk management. *Measures* - Peak HR - Peak VO2 - Resting BP - Framingham Risk Score - Anthropometry - Blood tests (HDL, LDL, total cholesterol) - Fasting blood glucose (HbA1C)	A graded maximal exercise test was used for all participants. - All anthropometric parameters were associated with the FRS - Strongest correlation with waist circumference - Important predictor variables for 10-year CVD risk (in order) - Waist circumference, Duration of injury, SAP, peak HR, level of injury, VO2 peak. *Conclusion:* Waist circumference is a simple practical measure of CVD risk.
Evans *et al*. (108), 2021 (South Africa)	Randomized controlled pilot study	Moderate quality	Chronic traumatic motor incomplete tetraplegia (*n* = 16)	Investigation of the effects of robotic locomotor training (RLT) vs activity-based training (ABT) over 24-weeks on cardiovascular changes *Measures:* - Brachial and ankle BP - HR - HRV - Cardiovascular efficiency during 4 physiological positions (supine at rest, standing at rest, 6 min arm ergometry, 6MWT)	*HR and BP* - RLT may be more effective than ABT in improving cardiac responses to orthostatic stress, particularly over a longer intervention period of 24 weeks. - Standing HR at 24 weeks was significantly lower in the RLT group (75.1 + −15.0 bpm) compared with the ABT group (95.6 + −12.6 bpm). - A postural tachycardia during standing is typical of individuals with SCI who are not able to elevate their blood pressure owing to associated autonomic dysfunction. - Brachial blood pressure did not change over the intervention but was elevated in comparison to similar studies. *HRV* - The stimulus of standing has comparable effects to RLT on the parasympathetic nervous system. - The stimulus of standing was comparable to exoskeleton walking during a 6MWT when evaluating HRV metrics as both physiological positions increased the sympathetic drive to the heart. *Cardiovascular efficiency* - The cardiovascular efficiency of exoskeleton walking improved, particularly over the first 6 weeks. - Both the RLT and ABT interventions were limited in their effect on brachial and ankle blood pressure. *Recommendation:* An RCT with a larger sample size is warranted to further examine these findings.
Yee *et al*. (6), 2021 (Canada)	Secondary analysis	Moderate quality	Secondary analysis of 3 studies of changes in HR during AD.	Participants had 24-hr BP monitoring.	- Median baseline SBP and HR lower in Cx than Tx SCI. - Median maximal SBP during AD comparable between Cx and Tx groups. - Total of 797 episodes of AD (SBP>20 mmHg), 74% with known trigger and 73% with unknown trigger. - AD episodes are associated with changes in HR: decreases in 59%, increases in 39%, and unchanged in 2%.
Dias *et al*. (109), 2021 (Brazil)	Cross-sectional study	Moderate-high quality	Adults with traumatic SCI (36 athletes, 52 non-athletes); 57 able-bodied individuals	Investigation of cardiac autonomic modulation - Heart rate variability (HRV) in the sitting position at rest and during a virtual reality (VR) game activity.	- Non-athletes with SCI had lower HRV with lower mean RR, SDNN, RMSSD, LF ms^2^, HF ms^2^, LH/HF ratio and pNN50 and higher mean HR compared to athletes with SCI and able-bodied controls. These parameters were comparable between athletes with SCI and able-bodied controls. - Cardiac autonomic modulation of athletes with SCI and able-bodied individuals was superior in both resting and physical activity conditions. There is acute cardiac autonomic adaptation with physical activity. - Increased drive of sympathetic activity with VR physical activity: increases in mean HR, SNS index and Stress index; decreases in global indices such as SDNN, LF ms^2^, the LF/HF ratio, SD2, and the SD2/SD1 ratio; withdrawal of parasympathetic activity (decrease in parasympathetic indices RMSSD, pNN50, HF ms^2^, and the PNS index) in athletes and able-bodied participants. *Limitations*: HRV findings between acute, subacute and chronic SCI patients were not classified.
Wecht *et al*. (110), 2022 (Canada)	Cross-sectional study	Moderate-high quality	Inpatients with SCI undergoing rehabilitation (*n* = 41).	Examination of the utility of the ISNCSCI and the recently revised ISAFSCI in documenting cardiovascular ANS impairment at a baseline assessment in patients with SCI during inpatient rehabilitation. *Measures*: - ISNCSCI - ISAFSCI - Beat-to-beat HR and BP Depth and rate of breathing	Passive re-positioning of patients into sitting by raining head of bed between 45° and 90° to test for OH. - No differences in supine HR or BP based on ISNCSCI or ISAFSCI assessments. - HF-HRV generally lower with more distal lesions. - No significant differences in HF-HRV or SBP in patients with or without ISAFSCI evidence of cardiovascular impairment. *Conclusion:* Neither the ISNCSCI nor ISAFSCI are sensitive to changes in ANS function following traumatic SCI.
Pino *et al*. (111), 2022 (USA)	Prospective cohort study	Moderate quality	Adult patients with SCI AIS A and AIS B; *n* = 14	Examination of safety and autonomic responses of the initial 14 participants recruited for the E-STAND clinical trial. - Automated tilt table testing - Continuous monitoring of BP and HR via finger photoplethysmography. - Epidural spinal cord stimulation (eSCS) was administered until blood pressure normalized and/or signs or symptoms of orthostatic intolerance were alleviated.	- From a cardiovascular standpoint, epidural SCS seems to be safe, with minimal adverse events observed across various stimulation parameters designed for cardiovascular control and motor function restoration. - During autonomic testing, prolonged (>30 s) elevations in SBP exceeding 150 mmHg were infrequently observed during epidural SCS (approx. 0.5% of cumulative epidural SCS time for all participants) and did not occur more frequently during epidural SCS compared to head-up tilt and supine conditions.
Soriano *et al*. (112), 2022 (Canada)	Observational study	Low-moderate quality	Chronic traumatic cervical SCI -C3–C7 (*n* = 12).	Assessment of the acute cardiorespiratory response and safety of passive leg cycling in people with tetraplegia - Passive leg cycling at 29 ± 1 rpm for 10 min using motorized cycle. - Arterial BP and HR measured with Finapres (stroke volume and cardiac contractility). - Cerebral blood velocity in the middle cerebral artery (MCA) and posterior cerebral artery (PCA) measured using Doppler probe. - Femoral artery diameter and flow-mediated dilation	- Passive leg cycling increases cardiorespiratory activity in people with cervical SCI, elevating BP, stroke volume, HR, cardiac output, flow-mediated dilation, and ventilation (one instance of AD). - Despite the rise in BP, cerebral blood velocity remained stable, resulting in reduced cerebrovascular conductance in both the MCA and PCA. - Leg cycling may be more beneficial than upper limb exercise alone, *Limitations*: HR is not a valid marker of exercise intensity in those with cervical SCI.
Samejima *et al*. (113), 2023 (Canada)	Case series		Chronic motor-complete SCI, (2 Cx, 1 Tx)	1. Investigation of the real-time impact of clinically approved eSCS in preventing DARS-induced AD in three individuals with Cx and Tx motor-complete SCI 2. Evaluation of the impact of eSCS on sympathetic nervous system activity during DARS *Measures:* - 24 h ABPM - Baseline BP and HR - During DARS, beat-by-beat BP and HR.	16-electrode array neurostimulator implanted between T10 and T12 (lumbosacral spinal cord). DARS was used to induce AD. - All 3 participants experienced episodes of AD during a daily 24 h period. - Participants 1 and 2 had no nocturnal dipping. - DARS without eSCS induced an elevation in SBP of greater than 20 mmHg and a simultaneous reduction in HR, indicative of AD: higher LF wavelet power concomitant with the elevation of SBP. *eSCS* - Prevented AD induced by DARS and decreased LF wavelet power in all participants. - Minimal changes in resting SBP, DBP and HR. - Active eSCS during DARS prevented AD (marginal elevation in SBP of less than the 20mmHg threshold for AD diagnosis, a minimal reduction in HR and decreased LF wavelet power). *Conclusion:* These observations provide evidence to support the use of eSCS to prevent AD episodes during routine bowel procedures.
Walter *et al*. (114), 2023 (Canada)	Prospective study	Moderate quality	12 participants with SCI and history of AD	Each participant underwent urodynamics (UDS) testing for autonomic dysreflexia (AD), 24-hour ambulatory blood pressure monitoring (ABPM), and assessments of urinary incontinence-related quality of life (QoL) both at baseline and during treatment. The treatment involved a 4-week supply of fesoterodine, with daily doses ranging from 4 to 8 mg, adjusted based on individual efficacy.	- The majority of participants experienced a reduction in the severity of autonomic dysreflexia (AD) during urodynamics (UDS). - The frequency of AD episodes during ambulatory blood pressure monitoring (ABPM) also decreased. - Additionally, there was a reduction in neurogenic detrusor overactivity (NDO), and urinary incontinence-related quality of life (QoL) improved. However, there were no significant changes in cognitive and bowel function. - Long-term effect of fesoterodine was not evaluated.
Boakye *et al*. (115), 2023 (USA)	Prospective study	Moderate-high quality	25 adults with motor complete chronic SCI, at least 2 years post injury	Three cohorts: Cohort 1a and 1b received step and stand training before and after implantation with epidural stimulation. Cohorts 2 and 3 received usual care before implantation, then underwent various sequences of cardiovascular (CV-scES), voluntary (Vol-scES), and stand stimulation (Stand-scES) training after implantation. All participants had scES surgery with a 16-electrode epidural paddle placed between the T11 and L1 vertebrae and an internal pulse generator (IPG)	- All participants gained voluntary control of lower limb movement and could independently regulate blood pressure compared to baseline. - Most patients reported meeting their expectations. However, the study's small participant number necessitates additional multicenter studies.
Huang *et al*. (116), 2024 (China)	Interventional study	Moderate quality	Patients with SCI at or above T6 with history of AD (*n* = 25).	Investigation of intravesical injection of BTX-A - 24-hr ambulatory BP monitoring used to record frequency of AD at baseline and 3 months post-injection - Urodynamic studies at baseline and 3 months - Change in SBP from baseline to maximum SBP during urodynamic studies.	- Maximum SBP and change in SBP decreased significantly after BTX-A injection - Frequency of bladder-related AD decreased significantly 3 months post-injection.

**Table 7:** Systematic/scoping review articles pertaining to assessment and management of cardiovascular autonomic function following SCI.

Author, year, country	Study design	CASP assessment	Topic	Outcomes and Conclusions
Warburton *et al*. (117), 2007 (Canada)	Systematic review 42 studies: BWSTT – 4 studies (*n* = 47) Arm exercise – 20 studies (*n* = 278) FES -assisted cycling – 18 studies (*n* = 233) Included RCTs and non-RCTs	Moderate-high quality	Investigation of the evidence to ameliorate risk of CVD in SCI	*BWSTT* - Discrepancy in findings of different studies - Level 4 evidence that BWSTT improves cardiac autonomic balance in incomplete tetraplegia - Level 4 evidence that BWSTT can lead to improvements in cardiac autonomic balance in a subset of people with motor complete SCI who respond with moderate to large increases in HR - Preliminary Level 4 evidence that BWSTT can improve arterial compliance in people with motor complete SCI *Upper extremity exercise* Level 1 evidence that: - Moderate intensity exercise 20–60 min/day, 3 days/week, for minimum 6 weeks improves cardiovascular fitness and exercise tolerance after SCI - Vigorous intensity (70%-80% HRR) exercise leads to greater improvements in aerobic capacity than moderate intensity exercise *FES-assisted cycling*: - Level 4 evidence that FES cycling performed for a min 3 days/week for 2 months improves musculoskeletal fitness, the oxidative potential of muscle, exercise tolerance and cardiovascular fitness *Glucose homeostasis*: - Level 1 and Level 4 evidence of improved glucose homeostasis following aerobic and FES training (20–30 min/day, 3 x per week for 8 weeks or more) *Lipid lipoprotein profiles and risk of CVD* - Level 1 evidence for the role of exercise (70% maximal HRR, 20 min/day, 3 x per week for 8 weeks) in reducing lipid lipoprotein profiles and risk of CVD
Krassioukov *et al*. (118), 2009 (Canada)	Systematic review 26 studies: 8 pharmacological (1 RCT), 21 (2 RCTs) non-pharmacological intervention studies	Moderate quality	Review of the evidence for the management of orthostatic hypotension (OH).	Small sample sizes (*n* = 1–7), except for one study with 231 participants. Key findings: *Pharmacological* - One low-quality pharmacologic RCT found midodrine effective for OH. - Level 4 evidence (single case series involving 2 patients), that fludrocortisone is not effective for OH - Level 5 evidence that daily ergotamine, combined with fludrocortisone, can successfully prevent symptomatic OH (single case study) - Level 5 evidence that ephedrine reduces the likelihood of a patient experiencing hypotension. - Level 5 evidence (single case study) that L-DOPS, in conjunction with salt supplementation may be effective in reducing OH. *Non-pharmacological* - 3 Level 2 RCTs and 6 non-RCTs support use of FES for OH. - Level 5 evidence that salt and fluid regulation in combination with pharmacological intervention may reduce symptoms of OH; however, no guidelines exist. - Level 2 evidence from a single low-quality RCT that pressure from elastic stockings and abdominal binders may improve cardiovascular physiological responses during sub-maximal, but not maximal, upper extremity exercises; however other studies contradict this. - Level 2 evidence that simultaneous upper extremity exercises may increase orthostatic tolerance during a progressive tilt exercise in individuals with paraplegia, but not tetraplegia. - Level 4 evidence that 6 months of BWSTT does not substantially improve orthostatic tolerance during a tilt test.
Krassioukov *et al*. (119), 2009 (Canada)	Systematic review 31 studies, 6 RCTs	Moderate-high quality	Review of the clinical evidence on strategies to prevent and manage autonomic dysreflexia (AD).	*Preventative strategies aimed at reducing common triggers for AD* - Level 4 evidence for effectiveness of Botulinum toxin into detrusor, intra-vesicular injection of capsaicin - Level 5 evidence that use of anticholinergics is not associated with a reduction in AD - Conflicting evidence for sacral denervation - Level 1 evidence for inter-sphincteric anal block with lidocaine limits AD response during anorectal procedures - Level 4 evidence for epidural anaesthesia during childbirth (vaginal or cesarean delivery) - Level 5 evidence that intra-operative hypertension may be reduced in susceptible patients - Level 1 evidence of no beneficial effect of topical anaesthetic to prevent AD during FES exercise *Management of acute AD* - Level 5 evidence that identifying possible triggers and decreasing afferent stimulation to the spinal cord is an effective non-pharmacological intervention - Level 1 evidence for use of nifedipine during cystoscopy or other diagnostic and therapeutic procedures - Level 5 evidence supporting use of nitrates - Level 4 evidence supporting use of captopril, Terazosin - Level 1 evidence for prazosin for prophylaxis - Conflicting evidence for use of phenoxybenzamine - Level 2 evidence that BP may be reduced using Prostaglandin E2 during electrical ejaculation - Level 2 evidence that sildenafil has no effect of BP during AD initiated by vibrostimulation
West *et al*. (120), 2012 (Canada)	Meta-analysis 98 studies (*n* = 1968)	High quality	Investigation of the effect of injury level on supine and seated CV changes after SCI	No significant between-group differences for age or time post-injury across lesion levels *Supine:* - SBP, DBP and HR lower in Cx compared with HT, LTL SCI and AB *Sitting* (only 2 studies): - SBP, DBP lower in Cx SCI compared with LTL and AB - HR different between Cx SCI and LTL SCI - Seated position only associated with lower SBP only in Cx SCI *Age and time post-injury*: - Significant positive associations between age and SBP in HT and AB, and age and DBP in HT only. - Time since injury positively associated with DBP in LTL. *Recommendation:* - BP should be measured in both supine and seated positions *Limitations:* - Most studies failed to document severity of SCI or grouped subjects with complete and incomplete injuries, very few studies with HT
West *et al*. (121), 2013 (Canada)	Systematic Review 21 studies – no RCTs	Moderate quality	Examination of the relationships between cardiovascular function and neurological and autonomic completeness of injury.	*Acute SCI*: - No clear consensus about whether resting HR, BP, or prevalence of BP abnormalities differs between neurologically complete and incomplete SCI. *Chronic SCI*: - 8 studies compared CVS function between individuals with neurological complete and incomplete chronic SCI, 2 of these also stratified their sample by autonomic completeness of injury. *Resting BP*: - 5 studies reported no difference in resting SBP or DBP between complete and incomplete injuries - One study reported that the diurnal rhythmicity in BP was intact in cervical incomplete SCI, but absent in 91% of cervical complete SCI - AD is more frequent in cervical complete than cervical incomplete SCI. -no differences in cardiorespiratory and metabolic indices between incomplete and complete SCI. - Only one study reported sitting and supine resting HR lower in cervical SCI than thoracic SCI *Heart rate variability:* - Investigated in 3 studies - Findings are controversial, however frequency component of HRV was increased at rest and in response to provocation in cervical incomplete compared to cervical complete SCI. - HF (parasympathetic) revealed no differences at rest between complete and incomplete Cx SCI.
Mills *et al*. (122), 2015 (Canada)	Systematic review 23 studies in SCI, 4 randomized crossover trials; 1 controlled crossover trial; 1 pre-post study	High quality	Review of non-pharmacologic treatment of orthostatic hypotension in a number of conditions, including SCI.	8 studies identified non-pharmacologic interventions for management of OH under 2 general categories: - Physical modalities (exercise, FES, compression, physical countermaneuvers, compression with physical countermaneuvers, sleeping with head up) - Dietary measures (water intake, meals). In SCI there is Level 1 evidence for: - FES in lower extremities improving orthostatic BP, standing time, and presyncopal symptoms in motor complete SCI during HUT. - Elastic abdominal binder (10% reduction in abdominal girth) decreasing prevalence of OH during active sitting and compression stockings for lower limbs. Level 2 evidence for: - Inflatable abdominal corset at 35mmHg or bilateral pneumatic leg splints at 65mmHg being significantly better than no device in maintaining BP during HUT. - For initial OH, lower-body muscle tensing for 40s after standing from squatting improving orthostatic BP and orthostatic symptoms Level 4 evidence for: - FES over bony prominences in lower extremities improving orthostatic BP and presyncopal symptoms in motor complete tetraplegia during HUT.
Lin *et al*. (123), 2022 (USA)	Systematic review 71 studies (*n* = 327); only 11 studies reported cardiovascular outcomes	Moderate quality	Assessment of the efficacy of epidural and transcutaneous spinal cord stimulation on recovery of function after SCI	*Cardiovascular outcomes:* 8 studies used eSCS and 3 used tSCS - Most common level of lead placement was T11–L1, shown to be effective in reducing OH; lead placement at T7–8 and L1–S1 were also found effective for addressing cardiovascular function. - The number of participants in each study ranged from 1- 6, and in one study, reported change in HR was an incidental finding - Both eSCS and tSCS have shown positive effects on autonomic cardiovascular function, especially in improving OH.
Chiou *et al*. (124), 2022 (USA)	Systematic review 18 studies (4 RCTs; *n* = 235); SCI between C4 and L3; one study reported only on shoulder pain;	Moderate-high quality	Efficacy of arm-crank exercise (ACE) on cardiorespiratory fitness in chronic SCI population	- Only 5 studies focused only on people with cervical injury; 15 studies reported peak oxygen consumption (VO_2_ peak) and peak power output after ACE to volitional exhaustion; significant increase in VO_2_ peak post-intervention; 10 studies reported a statistically significant increase in peak power after the ACE intervention. - Two studies measured forced vital capacity (FVC) and maximum voluntary ventilation (MVV) using spirometry; inconsistent findings - No strong correlation between magnitude of improvement in VO_2_ peak and total number of exercise sessions - Inconsistent findings regarding body composition and CVD risk factors
Laskin *et al*. (125), 2022 (Canada)	Scoping review 103 studies; most case studies or case series	High quality	Spinal cord stimulation for regaining motor, sensory and autonomic functions in patients with SCI.	- Review included: 55 epidural stimulation, 36 transcutaneous stimulation, 12 magnetic stimulation; incomplete stimulation parameters noted in many studies. - Primary focus on motor recovery. - 9 studies on epidural stimulation were specifically geared toward restoring bladder and bowel functions. - Cardiovascular outcomes were assessed as secondary measures (adverse effects of AD and hypotension); no studies prioritized cardiovascular autonomic functions as the primary outcome.
Flett *et al*. (126), 2022 (Canada)	Scoping review 19 eligible studies retrieved.	Moderate-high quality	Clinical electrical spinal stimulation research reporting effects on autonomic functions.	- Participant numbers low; mainly case reports or case series. - Early studies included autonomic outcomes as anecdotal, related to safety and tolerability. - Since 2018, 2 studies of TSCS and 6 studies of ESCS report planned systematic autonomic outcomes. - SCS at a variety of rostrocaudal spinal cord sites normalized BP regulation and temperature regulation - 4 studies of ESCS reported alterations in whole body metabolism, steady state or peak motor and exercise performance; lumbar ESCS increased VO2 Peak Other improvements noted: - Temperature regulation or reappearance of ability to sweat. - Increases in HR at rest; most changes in HR were either transient or highly variable - Normalized BP regulation at rest or while standing or stepping Although similar and multiple spinal neural structures are activated during SCS, very specific and individualized stimulation parameters and lead locations are necessary to influence either motor or sympathetic autonomic responses.

Notes: AB = able-bodied controls; ABPM = ambulatory blood pressure monitoring; ABT = activity-based training; ACE = angiotensin converting enzyme; AD = autonomic dysreflexia; ADFSCI = Autonomic Dysfunction Following Spinal Cord Injury; AIS = American Spinal Injury Association Impairment Scale; ANS = autonomic nervous system; BBB = blood brain barrier; BP = blood pressure; BPLF = low frequency component of blood pressure power spectrum; BPV = blood pressure variability; CBFV = cerebral blood flow variability; Cx = cervical SCI; DAP = diastolic arterial pressure; CRD-colorectal distension; DARS = digital anorectal stimulation; DBP = diastolic blood pressure; ECG = electrocardiogram; ELC = external leg compression; eSCS = epidural spinal cord stimulation; HDL = high density lipoprotein; HF = high frequency; HT = SCI between T1–T6; HR = heart rate; HRLF = low frequency component of heart rate power spectrum; HRHF = high frequency component of heart rate power spectrum; HRV = heart rate variability; HUT = head-up tilt; ISAFSCI = International Standards for the documentation of remaining Autonomic Function after Spinal Cord Injury; IWRF = International Wheelchair Rugby Federation; LDL = low density lipoprotein; LF = low frequency; LTL = SCI below T6; MAP = mean arterial pressure; MF = medium frequency; NE = norepinephrine; NGF = nerve growth factor; OH = orthostatic hypotension; pNN50 = percentage of RR intervals differing more than 50msec from the preceding one; PVS = penile vibrostimulation; PWD = P wave duration; RLT = robotic locomotor training; rMSSD = root mean square of the successive normal RR interval difference; RRI = interval between successive R-wave peaks; RVLM = rostro-ventro-lateral medulla; SAP = systolic arterial pressure; SBP = systolic blood pressure; SDNN = standard deviation of all normal RR intervals; SDNNi = mean of the SDs of all normal RR intervals for all 5-minute intervals; SPN = sympathetic preganglionic neurons; SSR = sympathetic skin response; Tx = thoracic SCI; tSCS = transcutaneous spinal cord stimulation; VCAM = vascular adhesion molecule; VLF = very low frequency; VO2 = peak oxygen consumption; BWSTT = Body weight supported treadmill training.

The majority of the studies reviewed emanated from Canada. Many of the narrative reviews reported similar findings with respect to the pathophysiology and clinical symptoms of cardiovascular autonomic function. Most of the evidence from systematic reviews, original research and case studies to date has been obtained in people with chronic SCI.

#### Cardiovascular autonomic complications and cardiovascular risk associated with SCI

The reviews have concluded that many cardiovascular autonomic complications persist into the chronic stages, the most common being low BP, OH, AD and arrhythmias (bradycardia and tachycardia). HR is lower in people with chronic cervical and high thoracic SCI than those with low thoracic SCI and able-bodied controls, and does not increase with exercise in those with high-level SCI.

BP is one of the most unstable cardiovascular parameters in people with high-level SCI. Resting BP is low, and this persists into the chronic phase. On shifting body posture to the upright position, BP falls further, resulting in OH. There appears to be no correlation between the severity of SCI and symptoms related to OH or AD. OH leads to an elevated risk of stroke and resting hypotension leads to impaired cognitive function. AD, characterized by episodic hypertension and reflex bradycardia, is quite common in people with chronic high-level SCI, and is potentially life-threatening. Complications include cerebral hemorrhage, cerebral ischemia or infarction, pulmonary oedema, cardiac arrest, cardiac arrhythmias, and silent myocardial ischemia (48). There are negative effects on cardiac performance and maximal cardiac output remains low after exercise.

Knowledge of the underlying pathophysiology has come from animal studies and involves disruption of the sympathetic pathways, alterations in the morphology of sympathetic pre-ganglionic neurons, plastic changes in spinal circuitry and end-organ maladaptation (arterial stiffness, and changes in heart structure and function) (15, 49). While parasympathetic control is usually preserved after SCI, there is a loss of the synergistic relationship between the sympathetic and parasympathetic control. With respect to OH, the loss of the skeletal muscle pump from the lower limbs is a contributing factor.

#### Measurement and documentation of autonomic dysfunction (Table 3)

An important milestone in relation to autonomic dysfunction after SCI occurred when an expert panel formulated definitions and classifications for autonomic dysfunctions following spinal cord injury (SCI) (67), followed by the development of the International Standards for the documentation of remaining Autonomic Function after Spinal Cord Injury (ISAFSCI) in 2012 (20). This has been recently revised and now includes guidance for standardized administration (70). The inter-rater reliability of the General Autonomic Function section was moderate to good (60); however, the construct validity of this section was found to be weak (63). Another questionnaire, the Autonomic Dysfunction Following Spinal Cord Injury questionnaire demonstrated a strong association between the self-reported total AD score and daily AD frequency with the number of AD events detected over a 24-hour period using ambulatory BP monitoring (17).

Orthostatic hypotension has been evaluated using the sit-up test or head-up tilt test. BP responses to the sit-up test were found to be reliable (96).

Assessment of sympathetic function has been done using a variety of techniques, including cutaneous vasoconstriction in response to somatosensory stimulation (56), the sympathetic skin response (SSR) (57), and the Valsalva maneuver (62). The most reliable interpretation of the SSR is whether or not it is present (94). However, this measure, used in isolation, may not be indicative of presence or absence of autonomic impairment, and a battery of tests is required to fully characterize autonomic dysfunction (56). In experimental studies, finger plethysmography has been used for beat-to-beat monitoring of BP. Measures of HR variability and BP variability have also been used to evaluate the various contributions of vagal and sympathetic mechanisms.

#### Management of cardiovascular autonomic dysfunction

Tables 4 and 5 summarize guidelines for managing and reducing cardiovascular risk and clinical management strategies for autonomic dysfunction. A clinical practice guideline, formulated by an international panel in 2021 (21), concluded that although there was good consensus among the expert panel, evidence-based recommendations for the management of autonomic dysfunction are limited, and based largely on expert opinion. There appears to be no single strategy for management used in clinical practice. Rather, a comprehensive approach combining preventive strategies, non-pharmacologic interventions, and pharmacologic management has been recommended. Newer options for managing autonomic dysfunction involve spinal cord stimulation, and these studies are summarized in Table 7.

#### Investigations of cardiovascular autonomic dysfunction

The original research studies and case reports summarized in Table 5 report cross-sectional and prospective investigations of changes in cardiovascular parameters with change in posture, wearing a harness, arm cycling exercise, robotic locomotor training, and cold application of the hand and foot, as well as complications over a 12-month period, P-wave dispersion, the effect of a cardiac pacemaker to restore normal heart rate, examination of diurnal BP patterns, pharmacological interventions to reduce AD severity associated with penile vibrostimulation, or bladder-related AD, the effectiveness of transcutaneous spinal cord stimulation in managing OH during an orthostatic challenge, inspiratory-expiratory respiratory motor training to reduce OH, and a psychometric investigation of the Autonomic Dysfunction Following SCI (ADFSCI) questionnaire.

The systematic reviews (Table 6), which were of moderate to high quality, include RCTs and non-RCTs. They report the evidence for the effectiveness of exercise in reducing cardiovascular risk, pharmacological and non-pharmacological interventions for the management of OH, preventative strategies for reducing triggers for AD, the relationship between injury level and supine and seated cardiovascular parameters, the relationship between cardiovascular function and neurological and autonomic completeness of SCI, and the efficacy of epidural and transcutaneous spinal cord stimulation in improving cardiovascular outcomes.

In recent years, novel studies with neuromodulation have shown positive results in stabilizing cardiovascular autonomic functions. There are, as yet, few studies and these have small sample sizes. Furthermore, there is significant variation in the level for application of spinal stimulation, as well as its intensity and the direction of current. Most studies have focused on the use of epidural spinal stimulation at or below the thoracic level. Most of the neuromodulation studies have measured cardiovascular results in a single intervention session or over a short-term.

## Discussion

The aim of this review was to identify research gaps and develop a comprehensive understanding of existing studies on the evaluation, measurement, and management of cardiovascular autonomic complications following SCI. The findings highlight a lack of a comprehensive research framework for autonomic function post-SCI and reveal several critical research gaps.

### Cardiovascular autonomic dysfunction

Cardiovascular autonomic complications following SCI are well-documented, with a higher prevalence in both acute and chronic stages and some overlap between the two groups (Table 2). However, there is a notable lack of evidence regarding their occurrence and characteristics during the subacute phase of SCI.

### Measurement or evaluation of cardiovascular autonomic dysfunction

Although the ISAFSCI is recommended for clinical practice alongside the ISNCSCI, its effective implementation requires training and experience. Concerns have been raised regarding its construct validity, and further validation is required. The tool includes several self-report questions, and clearer guidelines are needed regarding the appropriate time frame self-reporting symptoms of OH and AD (60, 63, 68). Its limited use in clinical and research settings highlights the need for refinement to improve its simplicity and broader applicability.

Another tool, the ADFSCI questionnaire has been found to provide a good indication of AD events, though not of hypotensive events (17).

Ambulatory BP monitoring (ABPM) is considered superior to clinical BP monitoring and provides the means to evaluate nocturnal BP as the most sensitive predictor of cardiovascular disease (58). While a few studies have used 24-hour ABPM to assess AD and OH (97, 99, 114), there is a significant gap in research regarding the characterization of ABPM responses during AD and OH events triggered by stressors. Physical and emotional stressors can provoke blood pressure fluctuations, potentially affecting the evaluation of AD and OH. Although some studies have controlled for alcohol and caffeine consumption on the test day and the day prior, general daily physical activities and emotional states have often been overlooked or not reported. Although participants with SCI may be asked to complete questionnaires or otherwise mark significant potential stressors (such as bowel routine), this is difficult in practice. Failure to account for activity levels or the intensity of stressors during the monitoring period may result in blood pressure changes that reflect variations in activity rather than true pathophysiological alterations. Furthermore, most of the primary research studies have evaluated CV parameters at rest or after a defined period of exercise or exertion (Table 3). No studies have assessed autonomic CV parameters during routine rehabilitation procedures in people with subacute SCI.

While CV dysfunction has been evaluated based on severity of injury, there appears to be a lack of agreement regarding the relationship of CV autonomic parameters with completeness or incompleteness of SCI.

### Management of cardiovascular autonomic dysfunction

The management of autonomic dysfunction following SCI is primarily guided by expert consensus-based clinical recommendations (Table 5). However, there is no definitive treatment for preventing or managing cardiovascular complications such as AD and OH. Further, existing management guidelines are largely based on low-quality evidence.

Novel strategies to reverse autonomic dysfunction, such as epidural spinal cord electrical neuromodulation, have demonstrated promising results, but to date, these have been performed in a small number of participants. Only a limited number of studies have evaluated the effects of transcutaneous spinal cord stimulation on CV autonomic parameters (101, 103, 123–126), particularly the use of cervical transcutaneous spinal cord neuromodulation (6, 106). Furthermore, relatively few studies have investigated 24-hour ABPM before and after spinal cord neuromodulation (6, 103, 113), especially with long-term neuromodulation.

## Conclusion

In summary, this review emphasizes the intricate nature and importance of addressing cardiovascular autonomic complications post-SCI. While certain complications are well-documented, gaps in research persist, particularly concerning sub-acute stages and standardized assessment tools. The chronic persistence of these complications underscores their impact on long-term health outcomes and risk of CVD. While promising methodologies like 24-hr ABPM and questionnaire-based assessment of CV autonomic function such as the ISAFSCI and ADFSCI exist, there is still room for improvement. Managing these complications remains challenging, necessitating a comprehensive approach blending preventive, non-pharmacologic, and pharmacologic interventions. Emerging strategies such as spinal neuromodulation show promise but require further research and clinical trials.

## Study limitations

This scoping review was limited to research in humans that was published in English. To maximize the quality of data extracted we excluded descriptions of protocols or proposed research, conference proceedings, abstracts, lectures, theses, editorials, and commentaries from this scoping review. By doing so we limited sources with incomplete data and minimized the risk of the duplication of data. Furthermore, studies were included only up to the last database search on 16 April 2024, without restrictions on commencement dates, which may have led to the exclusion of the most recently published studies.

## Supplementary Material

Supplemental.docx

## Data Availability

The authors confirm that the data supporting this study's findings are included in the article.
