# Supplementary material for: Evaluation and management of autonomic functions in patients with spinal cord injury: A scoping review
Source: J Spinal Cord Med. 2025 Apr 7;49(3):425–85. doi: 10.1080/10790268.2025.2485509 (PMC13123063; doi:10.1080/10790268.2025.2485509)

**Appendix A. Literature Search Strategies**

**Ovid MEDLINE(R) ALL <1946 to July 11, 2023>**

1 Spinal cord injur*.mp. 58983

2 tetrapleg*.mp. 4922

3 Quadripleg*.mp. 10941

4 Parapleg*.mp. 23909

5 Trauma*.mp. 503152

6 1 or 2 or 3 or 4 84945

7 (Trauma* adj3 (Spinal cord injur* or tetrapleg* or Quadripleg* or Parapleg*)).mp. 5154

8 autonomic.mp. 100191

9 cardio*.mp. 1155556

10 (hypertension or hypotension or high blood pressure or HBP).mp. 636175

11 (orthostat* or postur* or arrhythmi* or tachycard* or bradycard* or faint* or blackout or drop attack or heart rate or heartrate or pulse rate or HRV or pseudosyncop* or syncop* or tilt table or valsalva or cold pressure test or reflex*).mp. 753238

12 8 or 9 or 10 or 11 2242025

13 7 and 12 510

14 (intervent* or exercis* or management or treat* or physiotherap* or physical therap* or therap* or drug* or rehab* or pharmacol* or cellular or stimulat* or neuromodulation).mp. 16210570

15 13 and 14 412

16 (measur* or monitor* or tech* or data or metric* or score* or performance or outcome* or risk* or QoL or "quality of life" or morbid* or mortal* or questionnaire* or survey* or interview* or registry or registries).mp. 16822834

17 15 and 16 292

18 (study or studies or trials or observat* or cohort*).mp. 15079037

19 16 or 18 21935990

20 15 and 19 332

21 limit 20 to (human and english language) 235

<https://access.ovid.com/custom/redirector/index.html?dest=https://go.openathens.net/redirector/unimelb.edu.au?url=http://ovidsp.ovid.com/ovidweb.cgi?T=JS&NEWS=N&PAGE=main&SHAREDSEARCHID=57DsZtoEZtQ6R9ogBja4PHpgROJqhLk66CbRiB0LrLNma3L48gMWUxH5pBo1h76kD>

**Embase Classic+Embase <1947 to 2023 July 11>**

1 Spinal cord injur*.mp. 83379

2 tetrapleg*.mp. 7636

3 Quadripleg*.mp. 24280

4 Parapleg*.mp. 44849

5 Trauma*.mp. 684112

6 1 or 2 or 3 or 4 136204

7 (Trauma* adj3 (Spinal cord injur* or tetrapleg* or Quadripleg* or Parapleg*)).mp. 7289

8 autonomic.mp. 177903

9 cardio*.mp. 2120891

10 (hypertension or hypotension or high blood pressure or HBP).mp. 1338208

11 (orthostat* or postur* or arrhythmi* or tachycard* or bradycard* or faint* or blackout or drop attack or heart rate or heartrate or pulse rate or HRV or pseudosyncop* or syncop* or tilt table or valsalva or cold pressure test or reflex*).mp. 1266987

12 8 or 9 or 10 or 11 3950615

13 7 and 12 932

14 (intervent* or exercis* or management or treat* or physiotherap* or physical therap* or therap* or drug* or rehab* or pharmacol* or cellular or stimulat* or neuromodulation).mp. 25216426

15 13 and 14 783

16 (measur* or monitor* or tech* or data or metric* or score* or performance or outcome* or risk* or QoL or "quality of life" or morbid* or mortal* or questionnaire* or survey* or interview* or registry or registries).mp. 22086194

17 15 and 16 572

18 (study or studies or trials or observat* or cohort*).mp. 23969984

19 16 or 18 30945119

20 15 and 19 670

21 limit 20 to (human and english language) 528

<https://access.ovid.com/custom/redirector/index.html?dest=https://go.openathens.net/redirector/unimelb.edu.au?url=http://ovidsp.ovid.com/ovidweb.cgi?T=JS&NEWS=N&PAGE=main&SHAREDSEARCHID=61Tk4gYvei4T2pElgLHRnXtVIQCZWWRHP21gK7Uwnus1QAT7CSpX2maUpn4Zfj146>

**ALL COCHRANE**

EBM Reviews - Cochrane Database of Systematic Reviews <2005 to July 5, 2023>

EBM Reviews - ACP Journal Club <1991 to June 2023>

EBM Reviews - Database of Abstracts of Reviews of Effects <1st Quarter 2016>

EBM Reviews - Cochrane Clinical Answers <June 2023>

EBM Reviews - Cochrane Central Register of Controlled Trials <June 2023>

EBM Reviews - Cochrane Methodology Register <3rd Quarter 2012>

EBM Reviews - Health Technology Assessment <4th Quarter 2016>

EBM Reviews - NHS Economic Evaluation Database <1st Quarter 2016>

1 Spinal cord injur*.mp. 4578

2 tetrapleg*.mp. 412

3 Quadripleg*.mp. 617

4 Parapleg*.mp. 895

5 Trauma*.mp. 36041

6 1 or 2 or 3 or 4 5319

7 (Trauma* adj3 (Spinal cord injur* or tetrapleg* or Quadripleg* or Parapleg*)).mp. 447

8 autonomic.mp. 9409

9 cardio*.mp. 141188

10 (hypertension or hypotension or high blood pressure or HBP).mp. 93178

11 (orthostat* or postur* or arrhythmi* or tachycard* or bradycard* or faint* or blackout or drop attack or heart rate or heartrate or pulse rate or HRV or pseudosyncop* or syncop* or tilt table or valsalva or cold pressure test or reflex*).mp. 124632

12 8 or 9 or 10 or 11 295312

13 7 and 12 73

14 (intervent* or exercis* or management or treat* or physiotherap* or physical therap* or therap* or drug* or rehab* or pharmacol* or cellular or stimulat* or neuromodulation).mp. 1700073

15 13 and 14 73

16 (measur* or monitor* or tech* or data or metric* or score* or performance or outcome* or risk* or QoL or "quality of life" or morbid* or mortal* or questionnaire* or survey* or interview* or registry or registries).mp. 1510616

17 15 and 16 69

18 (study or studies or trials or observat* or cohort*).mp. 1616431

19 16 or 18 1863738

20 15 and 19 72

21 limit 20 to (human and english language) 72

<https://access.ovid.com/custom/redirector/index.html?dest=https://go.openathens.net/redirector/unimelb.edu.au?url=http://ovidsp.ovid.com/ovidweb.cgi?T=JS&NEWS=N&PAGE=main&SHAREDSEARCHID=1JZyyhiU3r6wIpEFq1OelwTW5R8hfPw4FxPxWT6kG9RkjtQzWT5EmjEo6sliYckNi>

**CINAHL DATABASE**

|  | Thursday, July 13, 2023 2:07:12 AM |
| --- | --- |

| **#** | **Query** | **Limiters/Expanders** | **Last Run Via** | **Results** |
| --- | --- | --- | --- | --- |
| S10 | ((S5 OR S6) AND (S4 AND S7 AND S8)) AND (S1 AND S4 AND S7 AND S8) | Narrow by Language: - english Search modes - Boolean/Phrase | Interface - EBSCOhost Research Databases Search Screen - Advanced Search Database - CINAHL Complete | 176 |
| S9 | ((S5 OR S6) AND (S4 AND S7 AND S8)) AND (S1 AND S4 AND S7 AND S8) | Search modes - Boolean/Phrase | Interface - EBSCOhost Research Databases Search Screen - Advanced Search Database - CINAHL Complete | 182 |
| S8 | S5 OR S6 | Search modes - Boolean/Phrase | Interface - EBSCOhost Research Databases Search Screen - Advanced Search Database - CINAHL Complete | 4,829,784 |
| S7 | S2 OR S3 | Search modes - Boolean/Phrase | Interface - EBSCOhost Research Databases Search Screen - Advanced Search Database - CINAHL Complete | 585,969 |
| S6 | (study or studies or trials or observat* or cohort*) | Search modes - Boolean/Phrase | Interface - EBSCOhost Research Databases Search Screen - Advanced Search Database - CINAHL Complete | 3,324,832 |
| S5 | (measur* or monitor* or tech* or data or metric* or score* or performance or outcome* or risk* or QoL or "quality of life" or morbid* or mortal* or questionnaire* or survey* or interview* or registry or registries) | Search modes - Boolean/Phrase | Interface - EBSCOhost Research Databases Search Screen - Advanced Search Database - CINAHL Complete | 4,175,596 |
| S4 | (intervent* or exercis* or management or treat* or physiotherap* or physical therap* or therap* or drug* or rehab* or pharmacol* or cellular or stimulat* or neuromodulation) | Search modes - Boolean/Phrase | Interface - EBSCOhost Research Databases Search Screen - Advanced Search Database - CINAHL Complete | 3,625,993 |
| S3 | (orthostat* or postur* or arrhythmi* or tachycard* or bradycard* or faint* or blackout or drop attack or heart rate or heartrate or pulse rate or HRV or pseudosyncop* or syncop* or tilt table or valsalva or cold pressure test OR reflex*) | Search modes - Boolean/Phrase | Interface - EBSCOhost Research Databases Search Screen - Advanced Search Database - CINAHL Complete | 182,212 |
| S2 | (autonomic OR cardio* OR hypertension or hypotension or high blood pressure or HBP) | Search modes - Boolean/Phrase | Interface - EBSCOhost Research Databases Search Screen - Advanced Search Database - CINAHL Complete | 455,250 |
| S1 | (spinal cord injury or sci or parapleg* or quadripleg* or tetrapleg*) N3 trauma* | Search modes - Boolean/Phrase | Interface - EBSCOhost Research Databases Search Screen - Advanced Search Database - CINAHL Complete | 2,772 |

Top of Form

| **SPORTDiscuss** | Saturday, July 22, 2023 12:50:20 PM |
| --- | --- |

| **#** | **Query** | **Limiters/Expanders** | **Last Run Via** | **Results** |
| --- | --- | --- | --- | --- |
| S1 | spinal cord injur* | Search modes - Boolean/Phrase | Interface - EBSCOhost Research Databases Search Screen - Advanced Search Database - SPORTDiscus with Full Text | 9,615 |
| S2 | tetrapleg* | Search modes - Boolean/Phrase | Interface - EBSCOhost Research Databases Search Screen - Advanced Search Database - SPORTDiscus with Full Text | 1,151 |
| S3 | (spinal cord injur* or sci or tetrapleg* or quadripleg* or parapleg*) N3 trauma* | Search modes - Boolean/Phrase | Interface - EBSCOhost Research Databases Search Screen - Advanced Search Database - SPORTDiscus with Full Text | 1,037 |
| S4 | (autonomic OR cardio* OR hypertension or hypotension or low blood pressure or high blood pressure or HBP) | Search modes - Boolean/Phrase | Interface - EBSCOhost Research Databases Search Screen - Advanced Search Database - SPORTDiscus with Full Text | 89,252 |
| S5 | (orthostat* or postur* or arrhythmi* or tachycard* or bradycard* or faint* or blackout or drop attack or heart rate or heartrate or pulse rate or HRV or pseudosyncop* or syncop* or tilt table or valsalva or cold pressure test OR reflex*) | Search modes - Boolean/Phrase | Interface - EBSCOhost Research Databases Search Screen - Advanced Search Database - SPORTDiscus with Full Text | 78,428 |
| S6 | (intervent* or exercis* or management or treat* or physiotherap* or physical therap* or therap* or drug* or rehab* or pharmacol* or cellular or stimulat* or neuromodulation) | Search modes - Boolean/Phrase | Interface - EBSCOhost Research Databases Search Screen - Advanced Search Database - SPORTDiscus with Full Text | 752,607 |
| S7 | (measur* or monitor* or tech* or data or metric* or score* or performance or outcome* or risk* or QoL or "quality of life" or morbid* or mortal* or questionnaire* or survey* or interview* or registry or registries) | Search modes - Boolean/Phrase | Interface - EBSCOhost Research Databases Search Screen - Advanced Search Database - SPORTDiscus with Full Text | 868,977 |
| S8 | (study or studies or trials or observat* or cohort*) | Search modes - Boolean/Phrase | Interface - EBSCOhost Research Databases Search Screen - Advanced Search Database - SPORTDiscus with Full Text | 564,191 |
| S9 | S4 or S5 | Search modes - Boolean/Phrase | Interface - EBSCOhost Research Databases Search Screen - Advanced Search Database - SPORTDiscus with Full Text | 147,380 |
| S10 | S7 or S8 | Search modes - Boolean/Phrase | Interface - EBSCOhost Research Databases Search Screen - Advanced Search Database - SPORTDiscus with Full Text | 1,037,725 |
| S11 | ((S7 OR S8) AND (S6 AND S9 AND S10)) AND (S3 AND S6 AND S9 AND S10) | Search modes - Boolean/Phrase | Interface - EBSCOhost Research Databases Search Screen - Advanced Search Database - SPORTDiscus with Full Text | 84 |
| S12 | ((S7 OR S8) AND (S6 AND S9 AND S10)) AND (S3 AND S6 AND S9 AND S10) | Limiters - English Abstract Available; Language: English Search modes - Boolean/Phrase | Interface - EBSCOhost Research Databases Search Screen - Advanced Search Database - SPORTDiscus with Full Text | 79 |

**PubMed database**

<https://pubmed.ncbi.nlm.nih.gov/advanced/>


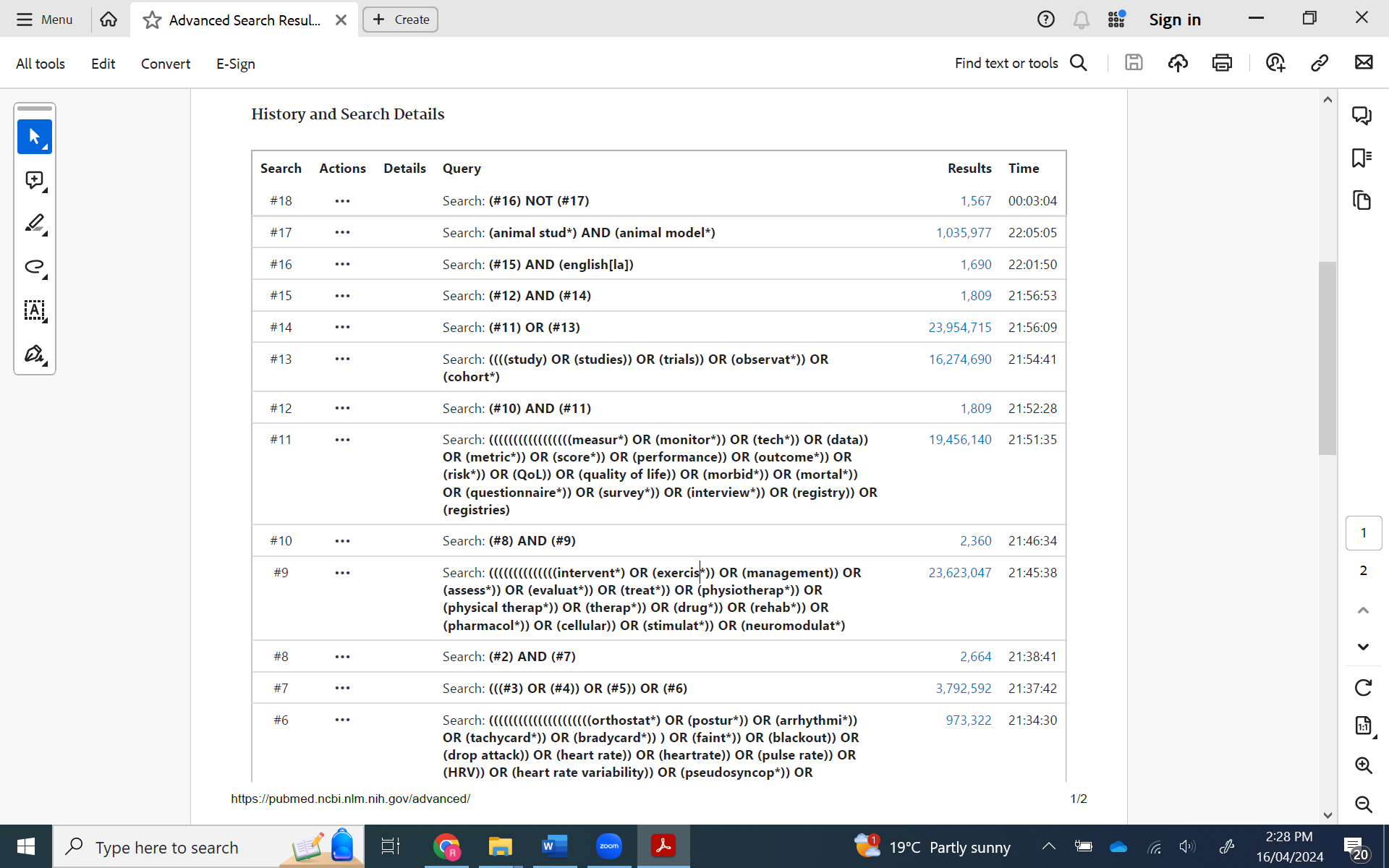

Supplement: Supplemental.docx [file YSCM_A_2485509_SM8820.docx]
